# Model-Based Analysis of Cell Cycle Responses to Dynamically Changing Environments

**DOI:** 10.1371/journal.pcbi.1004604

**Published:** 2016-01-07

**Authors:** Daniel D Seaton, J Krishnan

**Affiliations:** Department of Chemical Engineering, Centre for Process Systems Engineering, Institute for Systems and Synthetic Biology, Imperial College London, London, United Kingdom; Virginia Polytechnic Institute, UNITED STATES

## Abstract

Cell cycle progression is carefully coordinated with a cell’s intra- and extracellular environment. While some pathways have been identified that communicate information from the environment to the cell cycle, a systematic understanding of how this information is dynamically processed is lacking. We address this by performing dynamic sensitivity analysis of three mathematical models of the cell cycle in *Saccharomyces cerevisiae*. We demonstrate that these models make broadly consistent qualitative predictions about cell cycle progression under dynamically changing conditions. For example, it is shown that the models predict anticorrelated changes in cell size and cell cycle duration under different environments independently of the growth rate. This prediction is validated by comparison to available literature data. Other consistent patterns emerge, such as widespread nonmonotonic changes in cell size down generations in response to parameter changes. We extend our analysis by investigating glucose signalling to the cell cycle, showing that known regulation of Cln3 translation and Cln1,2 transcription by glucose is sufficient to explain the experimentally observed changes in cell cycle dynamics at different glucose concentrations. Together, these results provide a framework for understanding the complex responses the cell cycle is capable of producing in response to dynamic environments.

## Author Summary

The cell cycle is an exquisitely tuned process, alternating between states of cell growth, DNA replication, mitosis, and cytokinesis. While this process is robust, it is also responsive to diverse environmental signals. For example, cell cycle events may be delayed or advanced in response to changes in temperature or nutrient availability. While the molecular mechanisms underlying cell cycle progression have been well-characterised, how these mechanisms are perturbed by a cell’s environment is still not well understood. This problem is made difficult by the dynamically changing nature of the cell cycle itself. In this paper, we tackle this issue by performing a meta-analysis of mathematical models and experimental data describing cell cycle progression in the budding yeast, *Saccharomyces cerevisiae*. This shows how the timing of perturbations relative to the cell cycle stage (e.g. during DNA replication or mitosis) can give rise to qualitatively different responses. By looking for consistent patterns across multiple models and experimental datasets, we demonstrate how known molecular mechanisms change cell cycle behaviour in different nutrient conditions. This also allows us to make predictions for novel behaviours that can be experimentally tested in the future.

## Introduction

The cell cycle is the process by which cells alternate replication of their DNA with cell division. As a central process in the life of a cell, it is subject to multiple forms of regulation. These range from hormonal and growth factor signals in higher organisms, down to nutrient and stress signals in micro-organisms. While there has been much progress in understanding the mechanisms driving cell cycle progression, a system-level understanding of how signals regulate this progression has been lacking. In this paper, we investigate the dynamic response of the cell cycle to perturbations. In particular, we apply a combination of computational and mathematical analyses to study how the cell cycle of a particular model organism—the budding yeast *Saccharomyces cerevisiae*—responds to changes in conditions.

The progression of the cell cycle in *S. cerevisiae*, as in all eukaryotic cells, can be divided into four phases: the G1, S, G2, and M phases. The G1 and G2 (“gap”) phases mark the pauses between the essential processes of DNA duplication (which occurs during S phase) and segregation (which occurs during M phase). Several checkpoint mechanisms regulate progression through the cell cycle. These checkpoints ensure that progression through the cell cycle occurs only when the cell is in a suitable environment, and has adequately completed the previous stages of its cell cycle. For example, cells in G1 (with unreplicated DNA) must pass a checkpoint, regulated by factors such as nutrient availability and cell size, to go into S phase and begin synthesising DNA [1, 2]. Similarly, cells in the G2 phase must pass through checkpoints to enter mitosis (e.g. the spindle assembly checkpoint).

**Fig 1:**
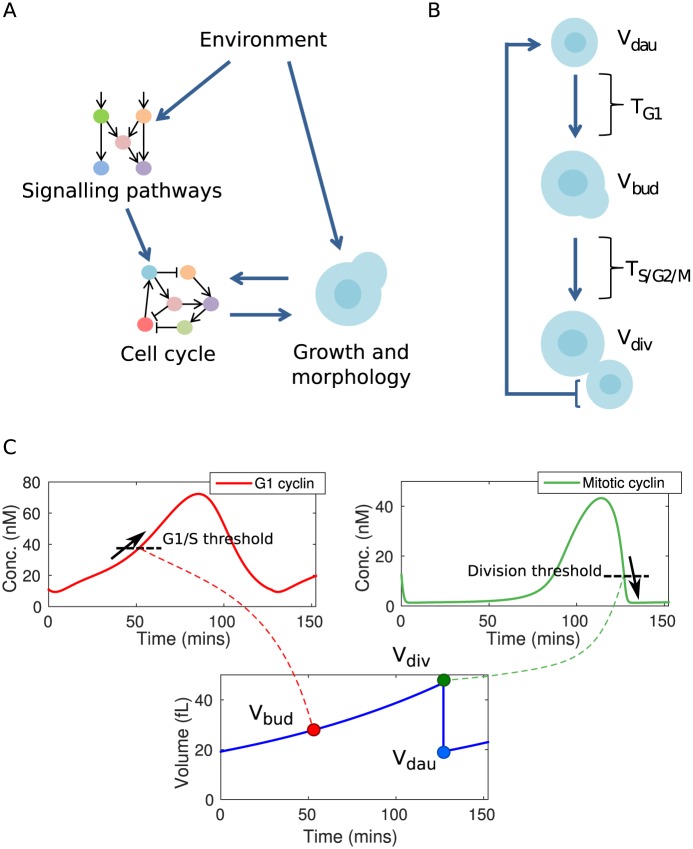
Overview of cell cycle dynamics in changing environments. (A) Schematic of environmental control of the cell cycle, involving direct regulation through signalling pathways and indirect effects through growth. (B) Cell cycle progression and growth. Cells grow isotropically for the duration of G1 (*T*_*G*1_), before polarising growth to a bud for the remainder of the cell cycle (*T*_*S*/*G*2/*M*_). The daughter cell size is determined by the bud size at division. (C) Simulation of cell cycle dynamics in the Barik model. Cell volume grows exponentially, while cell cycle components such as the G1 and mitotic cyclins oscillate. Budding occurs when G1 cyclins increase through a threshold, while mitosis occurs when mitotic cyclins decrease through a threshold.

In *S. cerevisiae*, progression through the cell cycle is coupled to changes in cell morphology and growth, as depicted in Fig 1. After birth, the cell grows isotropically during the G1 phase. The duration of this phase is strongly correlated with the size of the cell as a result of a “cell size checkpoint” [3]. Beyond this checkpoint, the cell is allowed to pass into S phase. Upon entry into S phase, DNA replication begins, the cytoskeleton is polarised, and a bud forms [4]. Cell growth continues, but with growth directed to the bud. The cell then passes through the G2 and M phases and begins the process of cytokinesis. This results in the bud splitting from the mother cell, producing a new daughter cell.

The prevailing view of the molecular mechanisms underlying cell cycle progression is one of interlocking positive and negative feedback loops which trigger a cascade of transitions in the appropriate sequence [5]. One of the central components in the cell cycle network is cyclin-dependent kinase (CDK), named Cdk1 in *S. cerevisiae*. A pre-requisite for the kinase activity of CDK during the cell cycle is the presence of cyclins. Different cyclins are expressed in different phases of the cell cycle and lend specificity to the CDK-cyclin complex, allowing regulation of many transcription factors and other processes [6, 7]. These cyclins may be broadly divided into different classes depending on the timing of their expression. For example, G1 cyclins are responsible for the transition from G1 into S phase, while mitotic cyclins are responsible for the transition from G2 into M phase. The abundance of cyclins is regulated at the levels of transcription, translation, and degradation. In addition, the CDK-cyclin complex may be rendered inactive by binding to a stoichiometric inhibitors such as Sic1 [8]. Ultimately, the cell cycle completes when CDK activity is reduced by the degradation of cyclins by the Anaphase Promoting Complex (APC) [9]. This allows the cell to progress through anaphase and cytokinesis. An illustration of this dynamic progression is shown in Fig 1C and 1D. As shown, the cell is born with low but increasing levels of G1 cyclins. When the level of G1 cyclin reaches a threshold, S-phase is initiated. Levels of G1 cyclins then decrease, with a complementary increase in mitotic cyclins maintaining Cdk activity. After sufficient time for progression through mitosis and the satisfaction of additional checkpoints, CDK inhibitors and components responsible for cyclin degradation (such as the APC) become active, along with the phosphatase Cdc14, which dephosphorylates Cdk substrates. This rapidly depletes CDK activity, allowing cytokinesis to occur and a new cell to be produced.

The distinct morphology of *S. cerevisiae*—in particular the correspondence between the initiation of S-phase and the appearance of the bud—means that it has been a useful model organism for the study of the cell cycle. A number of environmental cues have been found to regulate cell cycle progression in *S. cerevisiae*. For example, addition of glucose to cells growing in ethanol increases the average size of the cells at bud initiation and reduces the duration of the cell cycle [10]. The cell cycle is also responsive to changes in other nutrient signals [11–16], growth [1, 17, 18], osmotic stress [19, 20], and temperature [21]. In addition, under certain conditions the cell cycles of a population of cells can spontaneously exhibit partial synchronisation with an oxidative metabolic cycle [22, 23]. Finally, experiments in which cyclin expression is inducible by an external signal have demonstrated the possibility of mode locking the cell cycle to a periodic stimulus [24]. The responsiveness of the *S. cerevisiae* cell cycle to environmental conditions is a generic property of the eukaryotic cell cycle.

Despite the rapid accumulation of knowledge of the molecular details of the cell cycle mechanism and its regulation, such are the number of pathways and the complexity of the cell cycle itself that it is difficult to predict *a priori* how the system will respond to changes in conditions. As a result, it is also difficult to evaluate and interpret experimental observations and determine whether an observed phenomenon can be accounted for by known regulatory mechanisms. To this end, mathematical modelling approaches are useful to investigate hypotheses about cell cycle regulation. Models describing the dynamics of essential cell cycle components have existed for some time [25], and have reached high levels of molecular detail [26–31]. These models describe the interactions between key regulators of cell cycle progression, and formalise the understanding accumulated over decades of fundamental cell cycle research.

In this paper, a framework is developed for the investigation of the dynamic regulatory capabilities of cell cycle models, and by extension the cell cycle itself. This framework consists of exhaustive computational sensitivity analysis, allowing evaluation of how the cell cycle might respond to changes in conditions, both dynamically and after a sustained change in conditions. While the cell cycle is a highly nonlinear system, we note that similar approaches using sensitivity analysis of complex biological systems have been applied successfully before, e.g. in the study of circadian clocks [32, 33]. We apply this analysis to three models of the *S. cerevisiae* cell cycle [30, 34, 35]. This allows several key questions about cell cycle regulation to be addressed, focusing on understanding the interaction between the cell cycle and the key developmental transitions of *S. cerevisaie* (Fig 1). For example: to what extent can key cell cycle characteristics such as period and size at division be regulated independently? What qualitative behaviours can be observed in the response of the cell cycle to a sudden change in conditions? How flexible can this dynamic response be for a given eventual change in behaviour?

## Models

In this section, we describe the mathematical models under investigation and the parametric sensitivity analysis of these models. We begin with a basic phenomenological description of the budding yeast cell cycle, following [24]. This describes the phenomenology of cell cycle progression, rather than the biochemical details. Specifically, under some simple assumptions about the growth of the cell, it is possible to interrelate macroscopic cell cycle properties such as daughter cell size, cell cycle duration, and cell size at budding. This mathematical description then provides the orientation and basic framework for understanding the three detailed models that follow. These detailed models consist of ordinary differential equations (ODEs), and include both *S. cerevisiae*-specific models and a general model adapted here for use with budding yeast.

The underlying models of the regulatory network are coupled to this basic description of growth in two key ways, summarised in Fig 1. First, cell size affects cell cycle dynamics in each model by regulating the synthesis of molecular components. Second, the cell cycle dynamics determine the timing of budding and division through thresholds on the molecular components representing G1 and mitotic cyclins, respectively (Fig 1C). These interactions are described in detail for each model in S1 Text.

### Basic phenomenological description of the cell cycle

All of the models considered here share the same basic behaviour, with a continuously growing cell alternating between division and budding. The volume of the cell at budding and division, and the duration of cell cycle phases, constitute a simple description of the dynamics. Following [24], this model incorporates the assumptions that growth is exponential [3] (growing at an exponential rate *μ*), that all growth after budding is localised to the bud, and that the daughter cell receives all of the volume of the bud. The variables of interest are the cell cycle period of a daughter cell (i.e. the time from birth to division, denoted *T*_*div*_), the time from birth to budding (i.e. the duration of the G1 phase, denoted *T*_*G*1_), the size of the cell at division (denoted *V*_*div*_), at budding (denoted *V*_*bud*_), and the initial size of the daughter cell (i.e. the size of the daughter cell at birth, denoted *V*_*dau*_), and the fraction of the cell volume given to the daughter cell after division (denoted *f*). At constant growth rate, these variables are interrelated according to the following expressions:

$$\begin{array}{ccc} V_{div} & = & V_{dau}e^{\mu T_{div}}\\ V_{bud} & = & V_{dau}e^{\mu T_{G1}}\\ f & = & V_{dau}/V_{div} \end{array}$$ (1)

Note that the underlying molecular models control the timing of budding and division events, with the result that the fraction *f* is an emergent property of the models rather than a parameter. Similarly, *T*_*div*_ is determined by the dynamics of the underlying models, and is in general be different from the mass doubling time (MDT), *T*_*MDT*_, which depends only on *μ* (*T*_*MDT*_ = ln(2)/*μ*).

All models considered here give a pattern of behaviour that can be related directly to this simple description, after slight alteration to include a budding event where appropriate. The differences between the cell cycle models thus arise from the quantitative details of their structure and their parameter values. While more detailed models of the coupling between cell cycle to growth and metabolism have been suggested [36], the above description is an adequate minimal representation for the purposes of our investigation.

### Selection of suitable models to investigate

In this section, the models analysed are described, with model equations presented in S1 Text. The number of variables and parameters used in each model are also given. For more complete descriptions of these models, reference should be made to the corresponding papers. The three models are presented in order of increasing complexity, from a minimal model due to Pfeuty and Kaneko [34] (referred to here as the Pfeuty model), through a modified version of the Chen model [26, 35] (referred to here as the Chen model), and a more recent model incorporating detailed representations of multisite phosphorylation [30] (referred to here as the Barik model).

The simplified (Pfeuty) and detailed (Chen, Barik) molecular cell cycle models play complementary roles in the analysis. In particular, the simplified model demonstrates the range of behaviours possible with a minimal description of the molecular interactions. Thus, behaviours that are identified across all three models are unlikely to have arisen from a special combination of parameter choices and model structure. The detailed models, in turn, provide complementary insights as they contain explicit representations of important molecular regulators (e.g. cyclins). This allows specific hypotheses about regulation to be investigated (e.g. in the case of glucose signalling, below). The Chen and Barik models share several essential and well-established features with each other, for example the distinct roles of different cyclins in determining progression through different cell cycle checkpoints. In addition, the Barik model incorporates several additional mechanistic features that have been discovered more recently, such as the role played by Whi5 in progression through the G1/S transition [37–39].

All three models consist of systems of ODEs. This formalism is useful in this context as it provides a level of detail that allows investigation of how incremental changes in parameters change the system behaviour. Furthermore, a straightforward framework exists for the calculation and interpretation of sensitivity analysis of ODE models. It should further be noted that models that just consider particular phases of the cell cycle (e.g. models of the G1-S transition [20, 29] or mitosis [40, 41]) are not suitable for investigation here, since they cannot be run across multiple cell cycles.

#### The Pfeuty model

The Pfeuty model is the simplest model considered here, and was developed as a generic model of the eukaryotic cell cycle in order to study the coordination of growth with cell cycle progression [34]. This model is included in this investigation in order to give an idea of which cell cycle features can be captured by very simple models. The model includes 2 variables and 8 parameters. The two variables represent two quasi-molecular components, and are connected in a negative feedback loop. In this case, one variable represents components involved in the transition into S-phase. The activity of this component is repressed by a second component. This component, in turn, is responsible for initiating mitosis and cytokinesis. In order to simulate this model on a timescale appropriate for the *S. cerevisiae* cell cycle, parameters were uniformly rescaled according to the growth rate (see S1 Text).

#### The Chen model

The first detailed model of the budding yeast cell cycle considered is that of Chen et al [26] (more specifically, the moderately simplified version of this model considered in [35]), referred to here simply as the Chen model. This model brought together a large quantity of literature data to give a molecular cell cycle model that displayed the correct pattern of behaviour in the wild type, and in a large number (∼50) of cell cycle mutants.

This model contains multiple “hybrid” aspects, in which multiple events are controlled by concentrations of cell cycle components passing through specified checkpoints, at which point a rule is applied. These aspects make the original Chen model substantially different from the other models considered here. However, multiple simplifications of the Chen model were derived by Battogtokh et al [35] for the purpose of bifurcation analysis—the most complex variation is used here in order to represent the Chen model. This model includes 9 variables and 63 parameters.

#### The Barik model

The Barik model of the cell cycle was based upon the previous models of the Tyson group, with several modifications [30]. The model consists of mass-action kinetics, and as a result represents many more molecular species (e.g. in different phosphorylation states) than the other models. This was done so that stochastic simulations of the model could be performed to relate the noise characteristics of the model’s performance to experimental observations. This model includes 61 variables and 70 parameters.

### Sensitivity analysis

#### Steady-state sensitivity analysis

The sensitivity of an observable quantity, *Q*, to relative changes in a parameter, *k*, is defined by:

$$\begin{array}{c} C_{k}^{Q}=k\frac{dQ}{dk} \end{array}$$ (2)

It should be noted that this property is only defined for successions of daughter cells. This is because mother cells increase in mass at birth in each successive generation (both in reality [24], and in the above models). These sensitivities can be calculated by simulation, as described in S1 Text.

The quantity *Q* in Eq (2) can be any of several observable quantities, such as the relative phases of the peaks of different cyclins, the magnitude of the peak level of cyclin inhibitors, or the timing of cell cycle events such as kinetochore attachment. Such a general approach has been taken in the analysis of circadian rhythms [32, 42]. However, in the case of *S. cerevisiae*, as discussed above, many experiments on the behaviour of the cell cycle have concentrated on the changes in the timing of budding and division and their coordination with the cell’s size, and these are the characteristics investigated here (as defined and interrelated in Eq (1)). This connection to macroscopic, experimentally measured features motivates the specification of absolute rather than relative sensitivities of *Q* (as in [42]). Thus, the sensitivity coefficients have intuitive interpretations (e.g. $C_{k}^{T_{div}}=20$ implies a 20 minute change in *T*_*div*_ for a two-fold increase in *k*).

#### Dynamic sensitivity analysis

Dynamic sensitivity analysis of an oscillating system can be performed in a variety of ways, and can provide a variety of different types of information. The objective is to understand how the cell cycle models respond to step changes in parameters applied at different times during the cell cycle. This response is characterised by changes in the duration of the cell cycle, and the size of the cell at budding and division, over several generations. Sensitivity analysis has previously been applied to understand cell cycle dynamics, for example in identifying times at which these dynamics are unstable [43], and demonstrating common sensitivities of the dynamics of molecular components [44]. The present analysis complements these by linking the dynamics of cell cycle regulation to macroscopic phenotypes across multiple generations.

In general, the subsequent cycles following a perturbation may differ from both the initial cycle and the final cycle. For a cell cycle characteristic, *Q*, in the *i*th subsequent cycle following the application of the step change at time *t* (defined as the time since cell division), the dynamic sensitivity of *Q*_*i*_ to perturbations in parameter *k* is given by:

$$\begin{array}{c} S_{k}^{Q_{i}}\left(t\right)=k\frac{dQ_{i}}{dk}\left(t\right) \end{array}$$ (3)

The response dynamics to a step change in parameters are illustrated for the case of *V*_*div*_ and *T*_*div*_ responding to a step change in *k*_*s*,*bS*_ in the Barik model in S1 Fig. This shows how the transient response of the cell cycle to a step change in parameters at a particular time can be simulated across multiple subsequent generations, until the behaviour stabilises. This also provides a simple way of computing the dynamic sensitivities (i.e. by simulation), as described in S1 Text. It is also possible to represent the same information in the form of a sensitivity to perturbations of infinitesimal duration at time *t*, as given by:

$$\begin{array}{c} Z_{k}^{Q_{i}}\left(t\right)=\frac{dS_{k}^{Q_{i}}\left(t\right)}{dt} \end{array}$$ (4)

This gives an idea of how the function $S_{k}^{Q_{i}}\left(t\right)$ changes over time during the cell cycle. The dynamic sensitivities can be related to the sensitivities under constant conditions calculated in the previous section:

$$\begin{array}{c} C_{k}^{Q}=\lim_{n\to \infty }S_{k}^{Q_{n}}\left(t\right) \end{array}$$ (5)

A special case is the sensitivity of the cell cycle “phase” to changes in parameters. This represents the lasting changes in timing of cell cycle events following perturbations applied at different times [45]. For two cells under the same conditions, the cell cycle phase difference is equal to the difference in their timing of division. For two cells subjected to a change in parameters at different times, this phase difference is evaluated after the system has had sufficient time for transient changes to disappear. The sensitivity of phase to a step-change in parameters made at time *t*, defined relative to the phase when the perturbation is made at *t* = 0, is given by:

$$\begin{array}{c} S_{k}^{phase}\left(t\right)=\sum_{i=1}^{\infty }(S_{k}^{T_{div,i}}\left(t\right)-S_{k}^{T_{div,i}}\left(0\right)) \end{array}$$ (6)

As above (Eq 4), the instantaneous effect of a parameter change at time *t* is characterised by $Z_{k}^{phase}\left(t\right)$, given by:

$$\begin{array}{c} Z_{k}^{phase}\left(t\right)=\frac{dS_{k}^{phase}\left(t\right)}{dt} \end{array}$$ (7)

### Approximating changes in model behaviour using sensitivity analysis

Sensitivity analysis provides a straightforward way of understanding how combinations of parameter perturbations change cell cycle behaviour. In particular, we can approximate changes in behaviour (in the linear regime) by the linear combination of changes elicited by each perturbation, following [42]. For example, in the case of changes in *V*_*dau*_ in generation *i* following a perturbation in parameter *k* at time *t*, we have:

$$\begin{array}{c} \Delta V_{dau,i}\left(t\right)=\frac{\Delta k}{k}S_{k}^{V_{dau,i}}\left(t\right) \end{array}$$ (8)

Thus, for changes in multiple parameters *k*_1_, *k*_2_, …, *k*_*n*_, we have:

$$\begin{array}{c} \Delta V_{dau,i}=\sum_{j=1}^{n}\frac{\Delta k_{j}}{k_{j}}S_{k_{j}}^{V_{dau,i}}\left(t\right) \end{array}$$ (9)

An assessment of the accuracy of this approximation to changes in model behaviour away from the basal parameter set is shown for 8 parameters in the Barik model in S2 Fig. While the approximations are generally good, the highly non-linear nature of the model dynamics means that the range of parameter values for which this approximation is accurate is limited in some cases. However, even in these cases the qualitative changes in behaviour are matched across a wide range of parameter values. This demonstrates the utility of sensitivity analysis for understanding changes in model behaviour in a wide regime of parameter space.

## Results

### Steady-state sensitivity analysis identifies consistent model behaviours

We begin by evaluating the steady-state parameter sensitivities of the models, focussing on the macroscopic observable quantities such as the cell cycle duration (*T*_*div*_) and cell volume at division (*V*_*div*_). First, we note that, for a particular growth rate, the macroscopic cell cycle observables can be calculated in terms of only *T*_*div*_ and *V*_*div*_. For example, for *V*_*dau*_ and *T*_*G*1_:

$$\begin{array}{ccc} V_{dau} & = & V_{div}e^{-\mu T_{div}}\\ T_{G1} & = & -\frac{1}{\mu }\ln(\frac{V_{dau}}{\left(V_{div}-V_{dau}\right)}) \end{array}$$ (10)

**Fig 2:**
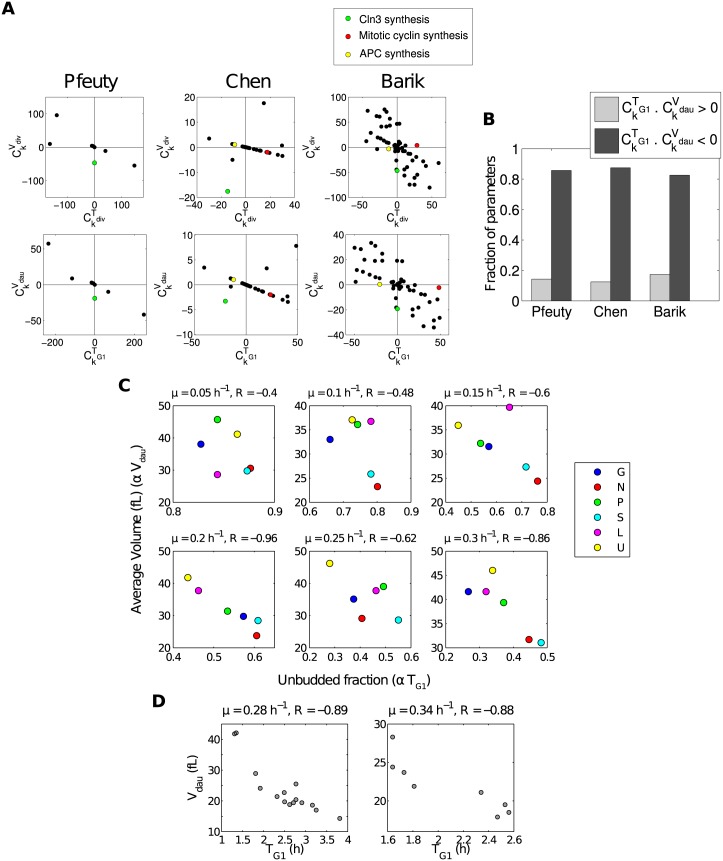
Consistent pattern of parameter sensitivities across models and experimental data. (A) Parameter sensitivities of *V*_*div*_, *V*_*dau*_, *T*_*div*_, and *T*_*G*1_ across all three models under constant conditions (i.e. basal parameter values). (B) Proportions of parameters for which the sensitivities of *V*_*dau*_ and *T*_*G*1_ have the same sign (light bars) and opposite sign (dark bars). (C) Negative correlation between average volume and unbudded fraction for cells grown at constant rate under different limiting nutrients (i.e. limiting glucose (G), nitrogen (N), phosphate (P), suphur (S), leucine (L), and uracil (U)). Data from [12]. (D) Negative correlation between *V*_*dau*_ and *T*_*G*1_ for cells grown at constant rate under different nutrient and genetic perturbations. Data from [46].

As a result, the sensitivity of the cell cycle to changes in parameters can be understood in terms of changes in *T*_*div*_ and *V*_*div*_ alone (or, equivalently *T*_*G*1_, *V*_*dau*_). This makes it natural to visualise the distribution of sensitivities in 2-dimensional scatter plots for each model, with each parameter shown as a point with position $\left(C_{k}^{T_{div}},C_{k}^{V_{div}}\right)$ (or, similarly, $\left(C_{k}^{T_{G1}},C_{k}^{V_{dau}}\right)$). This as shown in Fig 2A. This allows comparison across models of the properties of particular parameters, and identification of general trends across many parameters and models.

Some parameters of particular interest are those representing the regulation of cyclin synthesis and degradation. For example, the G1/S-specific cyclin Cln3 controls the timing of Start. Cln3 activity has been hypothesised to increase with cell size, and to therefore communicate cell size information to the cell cycle [47, 48]. Parameters representing the synthesis of Cln3 are present in both the Chen and Barik models, and an analogous parameter can be identified in the Pfeuty model (see S1 Text for details). As can be seen in Fig 2A, increasing the rate of synthesis of Cln3 acts to reduce the cell size in all three models, consistent with its role in cell size sensing. While changes in *V*_*div*_ are consistent across models, *T*_*div*_ is sensitive to changes in this parameter only in the Chen model.

Other species of interest are the mitotic cyclins. Mitotic cyclins increase through the G2-M transition, and are rapidly degraded by the APC upon exit from mitosis [49]. Parameters representing the synthesis of mitotic cyclins and the synthesis of APC subunits Cdc20 and Cdh1 are present in the Chen and Barik models (analogous components are not present in the Pfeuty model; see S1 Text for details). As can be seen in Fig 2, in both models these parameters act primarily to change *T*_*div*_ in opposing directions, with increased mitotic cyclin levels leading to a longer cell cycle period. While this is consistent across models, it should be noted that the changes in *V*_*div*_ predicted by the models are not.

Apart from the molecular species represented in the models, all three models also naturally include a parameter that specifies the growth rate (named *μ* by convention). In all three models, increasing the growth rate reduces the duration of the cell cycle, and increases the size of the daughter cell (S3 Fig), in agreement with experimental observations [10, 12, 50, 51]. This qualitative agreement has previously been noted for other cell cycle models [28]. In summary, it is clear that the models broadly agree on some, but not all, qualitative features of regulation by particular parameters.

Beyond specific parameters, it is also interesting to look at patterns observed across all parameters. It is clear from Fig 2A that in all three models most parameters act to modulate *V*_*dau*_ and *T*_*G*1_ in opposite directions (with a few clear exceptions in the case of the Chen model). This is quantified in Fig 2B. As a result, most combinations of parameter perturbations are expected to either increase *V*_*dau*_ and decrease *T*_*G*1_, or vice versa. This suggests that, for cells growing at the same rate under different conditions (i.e. with different environmental cues perturbing cell cycle components), *V*_*dau*_ and *T*_*G*1_ should be negatively correlated. A dataset that is useful for evaluating this model prediction was generated by Brauer *et al.* [12]. In their experiments, cells were grown in chemostats at 6 different growth rates (0.05, 0.1, 0.15, 0.2, 0.25, and 0.3 *h* ^−1^) under 6 different nutrient limitations (glucose, nitrogen, phosphate, sulphur, leucine, and uracil). Average cell volume (denoted $\bar{V}$, proportional to *V*_*dau*_) and the fraction of unbudded cells (denoted *F*_*G*1_, proportional to *T*_*G*1_ (see S1 Text for derivation)) were measured. Analysis of these data reveals a negative correlation between $\bar{V}$ and *F*_*G*1_ at all 6 growth rates, as shown in Fig 2C. Similarly, a recent study by Soma *et al.* measured *V*_*dau*_, *T*_*G*1_, and *μ* for various strains under different conditions [46]. Selecting those experiments for which *μ* was within a 0.02 *h* ^−1^ window, a clear negative correlation between *V*_*dau*_ and *T*_*G*1_ is again observed (Fig 2D). Finally, recently a high-throughput screen of cell cycle behaviour by Soifer *et al.* measured *V*_*dau*_ and *T*_*G*1_ in a range of mutants [52]. Considering only those mutants which were classified as having wild-type growth rates, this correlation was again observed (S4 Fig). The consistency of the qualitative behaviour of all three models with these experimental data suggests that they share essential dynamics that correctly describe cell cycle progression.

### Dynamic sensitivity analysis reveals complex responses to changing conditions

While the steady-state sensitivity analysis allows the characterisation of cell cycle models under constant conditions, it is also interesting to ask how the cell cycle responds to dynamic changes in parameters. Dynamic sensitivity analysis allows us to understand the complex dynamic behaviour which the cell cycle is capable of producing on its own. This provides a foundation for understanding how signalling networks with their own complex dynamics interface with the cell cycle.

**Fig 3:**
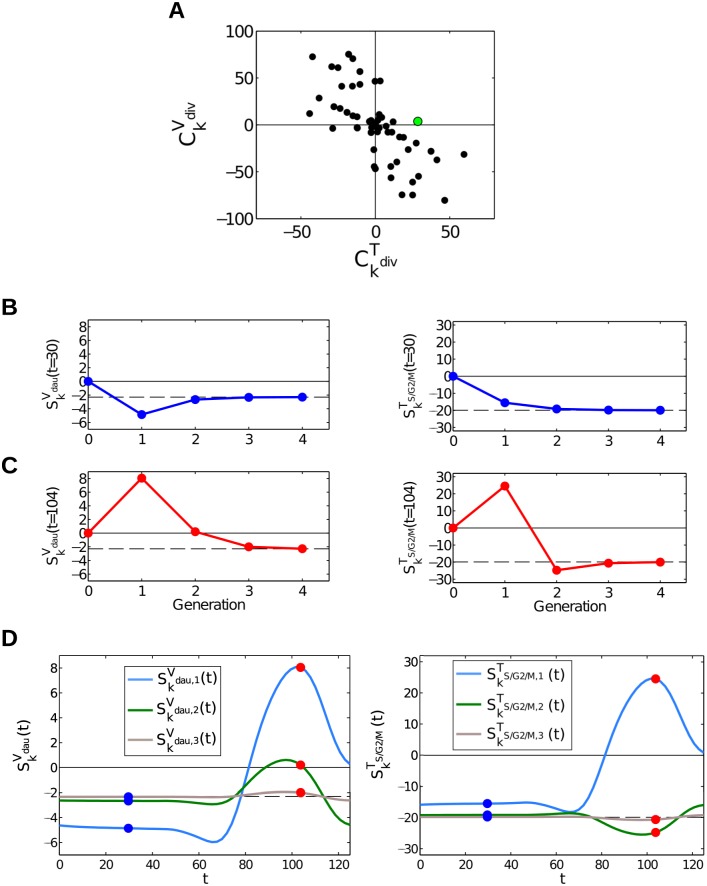
Example dynamic sensitivity analysis of the mitotic cyclin synthesis parameter in the Barik model (*k*_*s*,*bM*_). (A) $C_{k}^{T_{div}}$ and $C_{k}^{V_{div}}$ for all parameters in the Barik model, with the parameter representing mitotic cyclin synthesis marked in green. (B) Sensitivity of *V*_*dau*_ and *T*_*S*/*G*2/*M*_ for four generations following a step-change in parameters applied at 30mins. (C) As in (B), for a step-change in parameters applied at 104mins. (D) $S_{k}^{V_{dau}}\left(t\right)$ and $S_{k}^{T_{S/G2/M}}\left(t\right)$ down generations as functions of time. Points from (B,C) are marked by blue and red circles, respectively. Vertical dashed lines represent the time of the G1-S transition.

As detailed above, dynamic sensitivity can be characterised by the change in cell cycle characteristics down generations to a sustained step change in a parameter, starting at a particular time *t*. By way of example, the sensitivity of daughter cell size and the combined duration of the *S*/*G*2/*M* phases (*T*_*S*/*G*2/*M*_) to changes in the rate of synthesis of mitotic cyclin in the Barik model (specified by the parameter *k*_*s*,*bM*_) are shown in Fig 3. In this example, the sensitivity functions $S_{k}^{V_{dau}}\left(t\right)$ and $S_{k}^{T_{S/G2/M}}\left(t\right)$ are evaluated at two different times—one early (*t* = 30), and one late (*t* = 104) in the cell cycle (Fig 3B and 3C). This illustrates the changes in *V*_*dau*_ and *T*_*S*/*G*2/*M*_ that follow step changes made at these times. Two characteristics are apparent in this example, and are seen frequently in many parameters across all models: the dependence of the response on the timing of the perturbation, and the non-monotonic dynamics of this response. This sensitivity can also be visualised as a continuous function of the time of perturbation, as shown in Fig 3D.

As before, it is also instructive to consider the biological significance of this particular example. First, the qualitative characteristics of the response change depending on the time at which the perturbation is applied. Increasing mitotic cyclin synthesis early in the cell cycle reduces *T*_*S*/*G*2/*M*_ and *V*_*dau*_ in all subsequent generations, as compared to the initial state (Fig 3B). However, increasing mitotic cyclin synthesis at the end of the cell cycle increases *T*_*S*/*G*2/*M*_ and *V*_*dau*_ in the short term (Fig 3C). This can be understood by the role played by mitotic cyclins: their level must first increase to initiate mitosis, but must then decrease to allow the cell cycle to restart. Increasing mitotic cyclin synthesis at a time when cyclin levels need to decrease might be expected to temporarily delay cell cycle progression, as demonstrated by this sensitivity analysis. While this sensitivity analysis is qualitatively consistent with known biology, we note that an assessment of how mitotic cyclins drive the cell cycle in *S. cerevisiae* found that the models mis-predicted the quantitative extent of this sensitivity [53].

In summary, dynamic sensitivity analysis provides a useful tool for understanding the range of behaviours which the cell cycle is capable of producing. In all three models considered here, nontrivial dynamic behaviours were identified, including nonmonotonic changes in cell size down generations.

### The duration of the G1 phase is sensitive to parameter changes

It has been observed qualitatively in many studies that the duration of the G1 phase of the cell cycle is especially sensitive to changes in conditions. This manifests itself in a change in the fraction of unbudded cells in populations [10, 50]. It has also been observed that cells subjected to stresses transiently arrest the cell cycle at the G1/S-phase transition, without undergoing budding [54–58]. As a result, there has naturally been significant interest in understanding how signals determine progression through this transition. In this section, we investigate how the duration of the G1 phase changes under parameter perturbations of the models.

We begin by asking how changes in the duration of the pre-budded (*T*_*G*1_) and post-budded (*T*_*S*/*G*2/*M*_) phases of the cell cycle are related to one another in the phenomenological model (Eq 1). From this, we identify the relationship:

$$\begin{array}{c} C_{k}^{T_{S/G2/M}}=-fC_{k}^{T_{G1}} \end{array}$$ (11)

**Fig 4:**
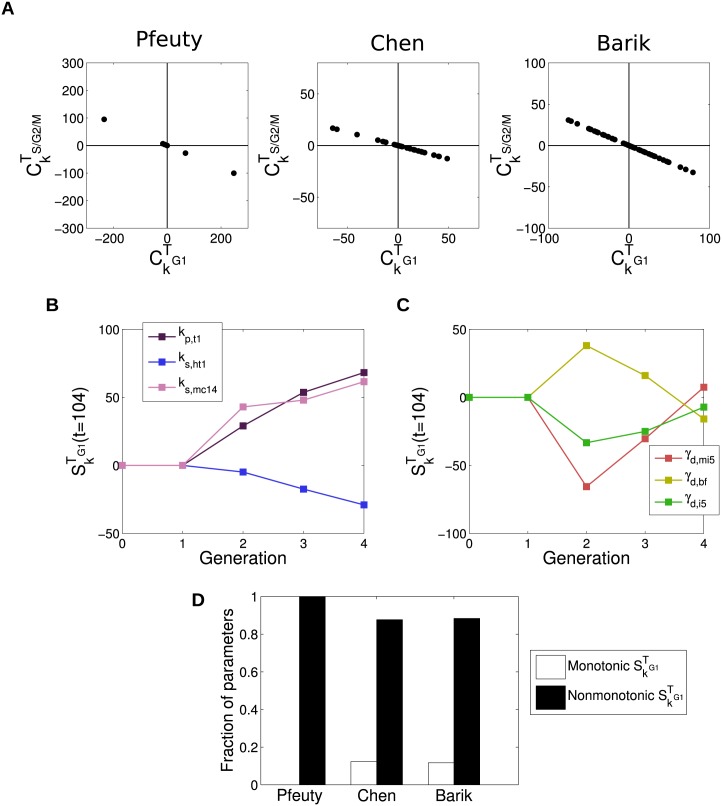
The duration of the G1 phase is particularly sensitive to changes in parameters. (A) Comparison of sensitivities of *T*_*G*1_ and *T*_*S*/*G*2/*M*_ to changes in parameters in constant conditions. The strict proportional relationship is clear in all cases. (B) Examples of monotonic changes in *T*_*G*1_ down generations. (C) Examples of nonmonotonic changes in *T*_*G*1_ down generations. (D) Comparison of fractions of parameters exhibiting monotonic and nonmonotonic changes in *T*_*G*1_ for all three models (see S1 Text for details of calculation).

Where *f* denotes the fraction of cell mass taken by the daughter cell upon division (see S1 Text for derivation). This demonstrates how parameter changes which alter the duration of the pre- and post-budded phases of the cell are fundamentally coupled to one another in the model. Furthermore, it shows that the magnitude of changes in the duration of the S/G2/M phases of the cell cycle are expected to be less than half the change in the duration of the G1 phase (since *f* ≤ 0.5, both *in silico* and *in vivo* [24]). This relationship is depicted for all three models in Fig 4A.

One counter-intuitive consequence of this is that changes in parameters affecting cell cycle progression during S/G2/M will modify *T*_*G*1_ more strongly than they modify *T*_*S*/*G*2/*M*_. Therefore, at steady-state, the duration of a particular phase of the cell cycle may be altered by perturbations that act during other phases. In particular, perturbations affecting processes during the G2/S/M phases will alter the duration of G1.

As discussed above, it is also commonly observed that moving cells into a stress condition can result in a transient accumulation of cells in G1 before the cell population eventually returns to its original state. At the single-cell level, this corresponds to a transient increase in *T*_*G*1_. One interpretation of this behaviour is that the cells take time adapt to the stress, during which cell cycle dynamics are perturbed, before the cells eventually return to their original state (and their original cell cycle behaviour). In the context of the analysis presented here, this would be analogous to changing model parameters for some time (while the cells are experiencing stress) before returning them to their original values (after the cells have adapted to the stress). However, we previously noted that a step-change in parameters can result in complex cell cycle dynamics, including transient changes away from the eventual behaviour. This was observed in the examples of $S_{k}^{T_{S/G2/M}}$ and $S_{k}^{V_{dau}}$ given previously (Fig 3), and is also true of changes in *T*_*G*1_. Since growth rate is held constant in these simulations, this behaviour is not the result of temporary changes in growth rate that might also be expected to accompany some changes in conditions. The prevalence of this behaviour can be quantified by calculating fraction of time which the sensitivity functions $S_{k}^{T_{G1,i}}\left(t\right)$ display a nonmonotonic sensitivity down generations, averaged across all parameters, with all models displaying at least 80% nonmontonic responses (Fig 4D). This suggests that transient responses of the cell cycle to changes in conditions must be interpreted with some caution. There are cases in which transient signalling appears to give rise to transient changes in cell cycle dynamics (e.g. [20]). However, the models suggest that transient signalling or changes in growth rate are not required for this behaviour to be observed.

In conclusion, these results demonstrate two causes for caution in the interpretation of changes in cell cycle dynamics in different conditions. First, that in cases where cells are grown under constant conditions, it is difficult to identify the cause for a change in cell cycle timing. This is because the duration of one cell cycle phase might change significantly as a result of regulation occurring during a different phase. Second, that transient changes in the duration of the G1 phase are a generic property of these models, and do not imply that signalling to the cell cycle is itself transient.

### Lasting changes in timing: Phase responses

The core yeast cell cycle oscillator interacts with other cellular oscillators, including the yeast metabolic cycle (YMC) [22], and is postulated to entrain slave oscillators such as oscillations in Cdc14 activity [59] and a transcriptional oscillator [60]. In addition, it is possible to partially mode-lock the cell cycle to an external periodic signal [24]. In other organisms, additional oscillator interactions have been identified, for example gating of cell cycle transitions by circadian clocks [61–63]. In this context, it is interesting to ask how dynamic perturbations alter the timing of cell cycle events. This has been investigated previously in the context of cell cycle responses to periodic forcing signals [64, 65]. Here, we are able to link control of cell cycle timing to the modulation of macroscopic cell cycle variables. The phase shift, Δ*ϕ*, resulting from a perturbation applied between the times *t*_1_ and *t*_2_ is given by its resultant effect on *T*_*div*_ down generations:

$$\begin{array}{c} \Delta \phi =\sum_{i=1}^{\infty }\left(T_{div,i}-T_{div,0}\right) \end{array}$$ (12)

This can also be calculated according to:

$$\begin{array}{c} \Delta \phi =\frac{\Delta k}{k}(S_{k}^{phase}\left(t_{1}\right)-S_{k}^{phase}\left(t_{2}\right)) \end{array}$$ (13)

This enables us to predict the mode-locking behaviour of the cell cycle to periodic forcing. For example, for a given periodic perturbation of the parameter *s*_*x*,2_ in the Pfeuty model, analogous to stimulating Cln3 synthesis, the phase response is predicted to mode-lock the cell cycle so that the stimulus occurs ∼39 minutes after cell birth (see S5 Fig). This prediction is borne out by simulations, with some error (∼7 minutes, S5 Fig).

**Fig 5:**
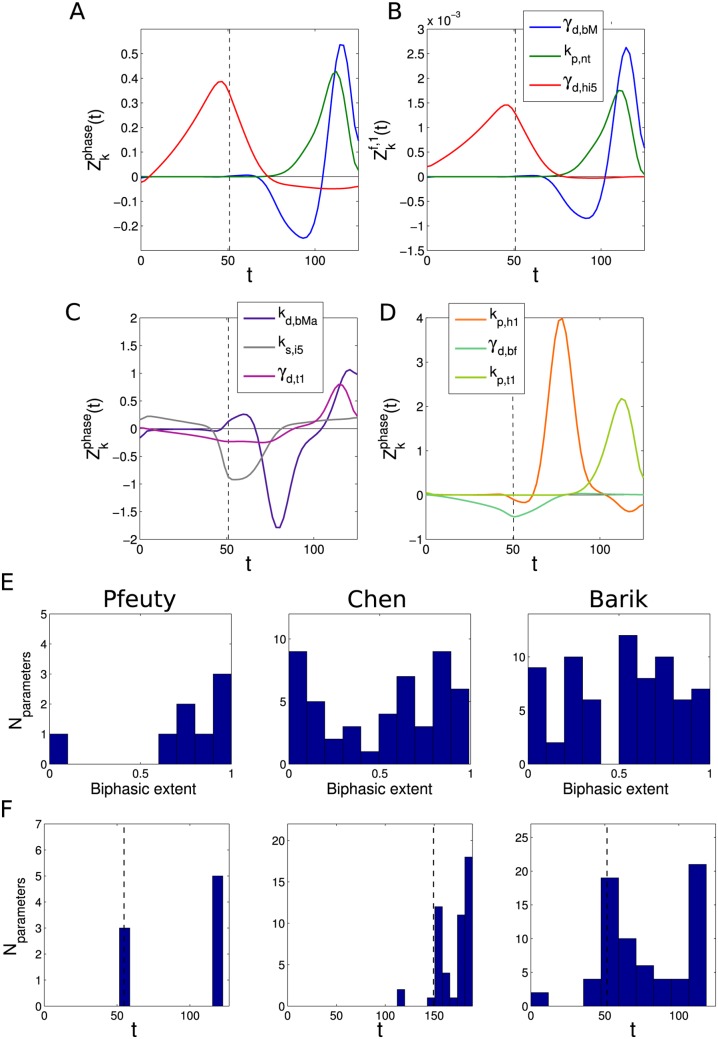
Consistent phase responses across models. (A) Phase responses for three exemplar parameters in the Barik model. (B) Sensitivity of the fraction of cell volume donated to the daughter cell for the three parameters shown in (A). The high similarity of the functions in (A) and (B) follows from the correspondence between phase shifts and daughter cell size fraction (Eq 14). (C,D) Examples of predominantly biphasic (C) and monophasic (D) phase response curves for parameters in the Barik model. (E) Distribution of biphasic extent of parameters for all three models, evaluated according to Eq (15). (F) Distribution of peak phase sensitivities for all three models. Vertical dashed lines represent the time of the G1/S transition.

The phase shift between two cells can be related to differences in the mass fraction donated to the daughter cell down generations. In particular, consider a perturbation which causes a temporary change in the fractions of mother cell volume donated to the daughter cell. Denote the initial fraction *f*_0_, and denote the deviation from this in the *i*th generation Δ*f*_*i*_. Then the phase shift is given by:

$$\begin{array}{c} \Delta \phi =\frac{1}{\mu }\ln(\prod_{i=1}^{\infty }\frac{f_{0}+\Delta f_{i}}{f_{0}}) \end{array}$$ (14)

(see S1 Text for derivation). In practice this limit converges rapidly (within a few generations). This establishes a link between how a parameter changes the mass of daughter cells, and how it changes the phase of the cell cycle. This correspondence is demonstrated in Fig 5A and 5B. We note that this is independent of any details of the models considered here, and applies to any asymmetrically dividing cell growing exponentially at a constant rate.

One notable qualitative feature of some of these phase response curves is that they are biphasic (i.e. both phase advances and delays are possible, depending on the timing of a perturbation). This property can be quantified for a parameter *k* by the metric *B*_*k*_:

$$\begin{array}{c} B_{k}=1-\frac{\left|\int_{0}^{T_{div}}Z_{k}^{phase}\left(t\right)dt\right|}{\int_{0}^{T_{div}}|Z_{k}^{phase}\left(t\right)|dt} \end{array}$$ (15)

This gives values of *B*_*k*_ ranging between 0 and 1. *B*_*k*_ is 0 for a completely monophasic pattern of sensitivity, as $Z_{k}^{phase}\left(t\right)$ is strictly positive or negative, so $|\int_{0}^{T_{div}}Z_{k}^{phase}\left(t\right)dt|=\int_{0}^{T_{div}}|Z_{k}^{phase}\left(t\right)|dt$. The distribution of *B*_*k*_ across the parameters of all models are shown in Fig 5E. From this, it is clear that many parameters in all models display this property. This is a property shared with other biological oscillators, for example circadian and neuronal oscillators [34, 66].

Another observation that can be made is that the phase shifts are most pronounced when perturbations are applied later in the cell cycle (from *T*_*G*1_ onwards). The distributions of the times of peak sensitivity of the parameters of all models are shown in Fig 5F. In all models there are two main groups of parameters—those peaking around *T*_*G*1_ and those peaking around *T*_*div*_—with very few parameters displaying peak sensitivity before *T*_*G*1_. This is somewhat counter-intuitive given the noted sensitivity of *T*_*G*1_ to parameter changes (see above). The robustness of the cell cycle model behaviour to perturbations during G1 has been observed previously in the case of the Chen model [43]. In summary, these results show that the cell cycle models consistently predict a preponderance of biphasic phase response curves, and further illustrate the qualitative differences in sensitivity observed before and after the G1-S transition.

### Case study: Glucose signalling to the cell cycle

The analysis presented above provides a framework for understanding the effects of perturbations on the dynamics of cell cycle progression. In order to demonstrate how the analysis presented can be applied to understanding signalling to the cell cycle, it is useful to consider a specific example. Here, we investigate how glucose-sensing signalling pathways might affect cell cycle progression. Glucose sensing is particularly important in this context, as the extra- and intracellular glucose levels are key determinants of nutrient availability. As such, several pathways have been identified through which glucose affects cell cycle components, both through direct sensing [13, 14, 16, 67, 68], and indirect effects via metabolism and growth rate [15, 16]. Here, we consider the effects of direct signalling pathways, and note that their effects can be separated from indirect, growth-rate-mediated effects in conditions where growth rate does not change in response to glucose levels. An example of this was recently demonstrated in experiments by Soma *et. al* in which changing glucose concentrations in the range of 0.05% to 2% had no effect on growth rate but did perturb the cell cycle [46].

**Fig 6:**
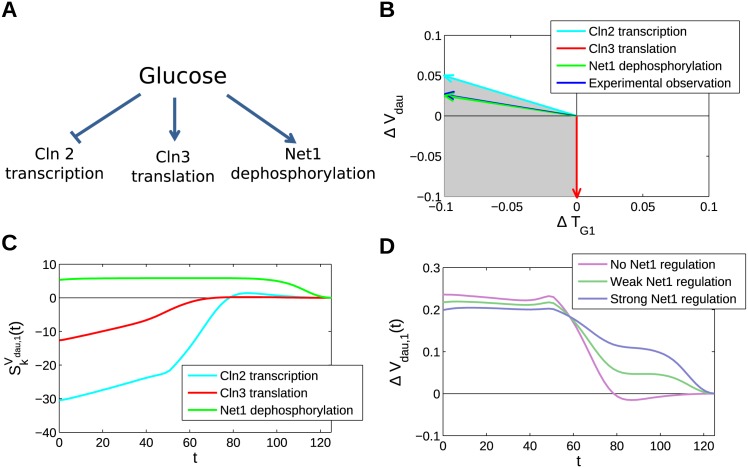
Glucose signalling case study. (A) A schematic of glucose regulation. Glucose is known to act on the cell cycle and many other processes through a diverse range of signalling pathways. We evaluate glucose modulation of the Barik model through regulation of Cln2 transcription (*k*_*s*,*mbS*_), Cln3 translation (*k*_*s*,*n*3_), and Net1 dephosphorylation (*k*_*d*,*t*1_, *k*_*d*,*nt*_). (B) The changes in behaviour mediated by increasing glucose levels are represented as vectors in the (Δ*T*_*G*1_, Δ*V*_*dau*_) space. The attainable range of behaviours is represented by the shaded region. This is consistent with experimental observations (dark blue line; see text for details). (C) The dynamic sensitivities of *V*_*dau*_ to the three coregulating pathways are shown. Note that the Net1 pathway is the only one that is capable of changing daughter cell size when parameters are changed late in the cell cycle (after ∼80 mins) (D) The change in daughter cell size in the first generation following an increase in glucose levels, as a function of the time of perturbation. The consequences of different balances of these three parameter perturbations are shown, all of which have the same eventual change in behaviour. The inclusion of strong regulation in mitosis (through Net1) allows dynamic response to changes in glucose levels late in the cell cycle.

We consider three particular forms of cell cycle regulation by glucose (Fig 6A). The first mechanism of cell cycle regulation by glucose involves the control of translation of Cln3—a cyclin responsible for inducing G1-S transition. The regulation of Cln3 translation is mediated in part through the direct regulation of the translation initiation factor eIF4E [69], and can also be controlled through the relief of competition for translation initiation factors (e.g. due to rapid degradation of GAL1 transcripts in the transition from galactose- to glucose-driven growth [70]). The rate of translation of Cln3 is represented in the Barik model by the parameter *k*_*s*,*n*3_. The second mechanism we consider is the repression of Cln2 expression by glucose [71]. In the Barik model, Cln2 falls within the class of G1 cyclins, denoted by ClbS. The rate of ClbS transcription is represented by the parameter *k*_*s*,*mbS*_. Finally, it is known that signalling through the TOR kinase complex is capable of modulating the activity of the PP2A phosphatase complex [72, 73]. Upon phosphorylation by the TOR1C complex, this phosphatase dephosphorylates a wide range of targets, including Net1 [74]. Net1, in turn, is responsible for sequestering the cell-cycle phosphatase Cdc14, which is required for progression through mitosis. The dephosphorylation of Net1 in the Barik model is represented by the constitutive activity of a generic phosphatase, Ht1. The model parameters representing this activity are *k*_*d*,*t*1_ and *k*_*d*,*nt*_, regulating free Net1 and Net1 in the RENT complex, respectively. A natural assumption is that regulation of this pair of parameters is coupled, and therefore that they are modulated proportionally to one another.

The above summary of some regulatory mechanisms is by no means complete, partly as a result of some regulatory components not being present in this model (e.g. the regulation of Cdk1 phosphorylation by Cdc25 and Swe1 [75]). However, since it includes components involved in regulating different cell cycle phases, it provides a useful starting point for understanding the range of behaviours that might be achieved by glucose regulation of the cell cycle. The effects of these regulatory mechanisms can be summarised by the following constraints on the parameter perturbations applied through this pathway (where the “signal”, assumed to be proportional to the availability of glucose, is represented by *G*, and the sensitivity of a parameter *k* to changes in *G* is denoted $R_{G}^{k}$):

$$\begin{array}{ccc} R_{G}^{k_{s,n3}} & = & \frac{1}{k_{s,n3}}\frac{dk_{s,n3}}{dG}>0\\ R_{G}^{k_{s,mbS}} & = & \frac{1}{k_{s,mbS}}\frac{dk_{s,mbS}}{dG}<0\\ R_{G}^{k_{d,nt}}=R_{G}^{k_{d,t1}} & = & \frac{1}{k_{d,t1}}\frac{dk_{d,t1}}{dG}=\frac{1}{k_{d,nt}}\frac{dk_{d,nt}}{dG}>0 \end{array}$$ (16)

These constraints imply a certain attainable range of responses in *V*_*dau*_ and *T*_*G*1_, meaning that only particular changes in *V*_*dau*_ and *T*_*G*1_ are possible in response to increases in *G*. For each parameter *k*, we can calculate contribution of that parameter to the changes in Δ*V*_*dau*_ and Δ*T*_*G*1_ that result from a change Δ*G*:

$$\begin{array}{c} (\begin{array}{c} \Delta T_{G1}\\ \Delta V_{dau} \end{array})=(\begin{array}{c} C_{k}^{T_{G1}}\\ C_{k}^{V_{dau}} \end{array})R_{G}^{k}\Delta G \end{array}$$ (17)

The responses possible in response to increasing glucose (Δ*G* > 0) can then be plotted as vectors in the (Δ*T*_*G*1_, Δ*V*_*dau*_) space for each pathway, as shown in Fig 6B. The shaded region represents the space spanned by linear, positive sums of these vectors, which is the attainable range of responses. Here, the regulatory mechanisms that we consider are limited to speeding up the cell cycle with increasing glucose levels (i.e. Δ*T*_*G*1_/Δ*G* < 0). Additionally, while this form of regulation can freely decrease the cell size without having a significant impact on the cell cycle period, there must be a decrease in period in order to effect an increase in cell size.

The consistency of the attainable region with experimental observations can be assessed by evaluating measured *V*_*dau*_ and *T*_*G*1_ values under different glucose concentrations and constant growth rate, as reported in [46]. The linear correlation between these values at three glucose concentrations (0.05, 0.1, and 2%) suggest the following empirical relationship, as depicted in S6 Fig (note that *V*_*dau*_, *T*_*G*1_ are in units of fL and minutes, respectively):

$$\begin{array}{c} \Delta V_{dau}=-0.27\Delta T_{G1} \end{array}$$ (18)

Note that this makes no assumption about the explicit relationship between glucose levels and the magnitude of parameter perturbations. The corresponding sensitivity to changes in glucose is then given by:

$$\begin{array}{c} \left(\begin{array}{c} \Delta V_{dau}\\ \Delta T_{G1} \end{array})\propto (\begin{array}{c} -0.27\\ 1 \end{array}\right) \end{array}$$ (19)

As shown in Fig 6B, this lies within the attainable region, confirming that this simple combination of regulations is consistent with the observed changes in behaviour.

An interesting aspect of the attainable region is that it is bounded by the opposing effects of stimulation of Cln3 translation and inhibition of ClbS transcription by glucose. This means that regulation of Net1 dephosphorylation does not broaden the range of behaviours that can be brought about through the pathway under constant conditions. Additionally, we observe that any change in behaviour (Δ*T*_*G*1_, Δ*V*_*dau*_) within the attainable region can be achieved in an infinite number of ways depending on the relative strengths of the three posited regulatory mechanisms (see S1 Text and S7 Fig). These different combinations of parameter perturbations will, by construction, have identical cell cycle behaviour under constant conditions, but may have distinct behaviours under dynamic changes in conditions.

In order to evaluate the potential for diverse dynamics in this system, we fix the change in behaviour achieved by parameter perturbations according to experimental observations (Eq 19), and consider three cases: no, weak, and strong up-regulation of Net1 dephosphorylation with increasing glucose levels. These changes are automatically balanced by changes in Cln3 and ClbS regulation by the constraint to achieve the specified (Δ*T*_*G*1_, Δ*V*_*dau*_). The resultant changes in the dynamic sensitivity ($S_{k}^{V_{dau,1}}\left(t\right)$) shown in Fig 6D are the result of differences in the timing of sensitivity of the cell cycle to the different parameters (see Fig 6C for the individual sensitivity profiles). Regulation of Cln2 transcription and Cln3 translation alone is only capable of modulating cell cycle progression around the G1-S transition, while regulation of Net1 dephosphorylation modulates progression through mitosis. Therefore, regulation of Net1 in the model allows for a faster response to changes in glucose levels by extending the time window of responsiveness to glucose levels.

It has been noted previously that glucose levels act predominantly to modulate duration of the G1 phase of the cell cycle [76], as discussed above in the more general case. An important conclusion arising from the work presented here is that this form of regulation does not exclude active regulation of processes occurring during mitosis (or other phases of the cell cycle). Indeed, as long as counteracting pathways can be modulated in tandem, regulation of processes occurring in mitosis may be a useful strategy for dynamic adjustment of cell cycle characteristics after a change in conditions. In the particular example of strong Net1 regulation shown in Fig 6D, this is seen to lead to a more rapid modulation of *V*_*dau*_ than would be possible if only Cln2 and Cln3 were regulated. As discussed above, observations of cell populations under constant conditions (e.g. the chemostat experiments in [12, 76]) are not capable of distinguishing between these strategies of regulation.

In summary, this analysis demonstrates that control of the G1/S transition is insufficient for rapid adjustment of the cell cycle to changing conditions. In order for rapid response to changing conditions, it is necessary for the components that are active during the S/G2/M phases of the cell cycle to be regulated by environmental signals. Furthermore, the effects of such perturbations may only be observable in experiments in which response dynamics are observed. Cell cycle sensitivity during mitosis has been observed experimentally in response to sudden nutrient starvation or application of rapamycin [77, 78], suggesting that investigation of nutrient signalling under constant conditions can indeed mask important regulation.

## Discussion

Cell cycle progression is a highly regulated process. This is a result of the importance of the processes it coordinates, and of the fine-tuned response required in changing conditions. A number of environmental stimuli have been observed to regulate cell cycle progression [16], and in some cases regulatory components have been identified. While mathematical models have been able to provide insight into cell cycle responses to some particular environmental changes (e.g. in the case of osmotic stress [20]), a broader view of how the cell cycle regulatory network might respond to environmental changes, and how that might affect subsequent growth patterns, has been lacking. It is clear that this is a problem of importance in both basic and applied contexts and that its analysis requires a systematic approach.

Our analysis was focussed on three mathematical models spanning a range from the simple (the Pfeuty model [34]), to the complex (the Chen [26, 35] and Barik [30] models). This revealed that some patterns of sensitivity were common to all models. For example, an anticorrelation between changes in G1 phase duration and daughter cell size was observed in all three models, and matched experimental observations [12, 46, 52]. These are also reminiscent of correlations observed at the single-cell level within populations of cells [3]. In addition, the models were shown to exhibit other qualitative behaviours that are observed experimentally, such as a sensitivity of G1 phase duration to perturbations. The consistency of model behaviours with experimental observations demonstrates that the models capture essential properties of cell cycle behaviour beyond those typically considered (e.g. the behaviour of cell cycle mutants [26]). The fact that that these behaviours are observed even in the simplified Pfeuty model suggests that they are robust features of the cell cycle. This suggests that other aspects of model behaviours identified here, such as the prevalence of biphasic phase response curves, are good candidates for further investigation.

Sensitivity analysis characterises changes in model behaviour in response to small perturbations, providing a platform for understanding their behaviour under large perturbations that may elicit nonlinear responses. However, it is important to recognise that changes that result in bifurcations which transform the qualitative behaviour must be analysed with tools from bifurcation theory. This constitutes an important class of cell cycle behaviours, including cell cycle arrest, meiosis, or the transition to endoreplication. Bifurcation analysis has been applied to understand these behaviours in a variety of cases (e.g. [79]), and provides insights into system behaviour that are complementary to those obtained by sensitivity analysis. Bifurcation analysis has also been an vital tool for understanding the dramatic changes in cell cycle behaviour caused by loss of some cell cycle genes (e.g. [28]). Through a combination of sensitivity analysis in the linear regime, and bifurcation analysis and simulations in the nonlinear regime, a comprehensive analysis of cell cycle behaviour in dynamic environments can be undertaken.

While this study has focussed on *S. cerevisiae* as a model system in which to study the cell cycle, related questions arise in a range of contexts of both fundamental and applied interest. For example, the question of how environmental cues regulate cell cycle progression is a general one, and is of interest in other yeast species, as well as in plant and animal systems. Though the cell cycle mechanisms are somewhat different in these systems, a similar approach to that taken here can be used to address this class of questions. In an applied context, having a mechanistic basis for understanding the connections between extracellular conditions, growth, and cell cycle progression in yeast is an important practical tool, for example in maximising yield of a valuable product.

The relevance of parameter sensitivity analysis to experimental studies of cell cycle behaviour depends on technology for accurate observation of cellular behaviour, fine control of cellular environment, and manipulation of cellular network structures. Rapid advances in microfluidic and imaging technology are addressing these issues [39, 52, 80–82], with current methods capable of observing hundreds of cells over many generations under rapidly changing conditions [83]. The ability to measure coordinated changes in regulatory network and cell cycle dynamics in response to perturbations is allowing increasingly detailed understanding of molecular mechanisms (e.g. [78]). This then makes it feasible to perform controlled changes in the environment and observe the resulting macroscopic changes in growth patterns. In addition, the burgeoning possibilities of synthetic approaches allow hypotheses about molecular mechanisms to be explored at unprecedented levels of detail [84]. The combination of quantitative modelling methodologies such as those employed here with these high-throughput, quantitative experimental approaches will allow for a significant improvement in our understanding of cell cycle and growth progression in varying environments.

Overall, by performing systematic steady state and dynamic sensitivity analysis to a range of detailed and simplified models, we have established a methodological platform to investigate the effects of dynamically varying environments on the cell cycle. Future extensions may incorporate bifurcation analysis to understand qualitative transformations in behaviour in the nonlinear regime. While we have applied our analysis to understand characteristics such as cell size and cell cycle duration in this particular study, it can also be applied to understand other characteristics of cell cycle behaviour. In conjunction with experiments, this approach provides a sound basis for beginning to understand the roles of the different parts of the cell cycle machinery in generating these responses. Furthermore, it also provides a basis for developing simplified descriptions which combine biological realism and mathematical soundness. This may be important in application domains. Finally, this approach provides a new window into the cell cycle as a complex system, and a route into understanding how dynamic information processing is undertaken by the cell cycle control system.

Mathematical models of the cell cycle have been useful in describing how known molecular interactions give rise to the observed complex dynamics [85], and to predict the behaviour of cell cycle mutants [86]. Despite the fact that these models may contain many parameters, they exhibit a fairly limited range of behaviours. This arises from the fact that these models encode similar regulatory logic. As a result, while our understanding of the biochemical details may change substantially, if the regulatory logic is broadly the same, we expect future mathematical models to exhibit similar behaviours. We further note that, if we focus on particular macroscopic, experimentally observable features (as is the case here), the range of behaviours for these features is especially restricted. A reduced effective dimensionality has been noted in a range of biological models, including models of the cell cycle [42, 87, 88]. Overall we find that the models are capable of reproducing a range of experimental observations. This consistency in the face of considerable molecular and dynamic complexity suggests that these models will be valuable tools for understanding how the cell cycle responds to changing environments and for utilizing this in multiple applications.

## Supporting Information

S1 Text Supplementary Information. (PDF)

S1 Fig Simulating the dynamic response of the cell cycle of a step-change in parameters. The parameter *k*_*s*,*bS*_ in the Barik model undergoes a step-change (A), which changes the volume at division (*V*_*div*_) and cell cycle duration (*T*_*div*_) in subsequent generations (B).
(EPS)

S2 Fig Qualitative assessment of approximation of model behaviour by extrapolation of local sensitivity in the case of the Barik model. Eight different parameters were increased by 20% at different times during the simulation, with the *V*_*div*_ in the first generation compared to an estimate based on the sensitivity analysis.
(EPS)

S3 Fig Model sensitivity to changes in the growth rate parameter, *μ*. In all three models, increasing growth rate leads to larger daughter cells and a reduced duration of G1 (upper panels), consistent with experimental observations. In addition, the models consistently predict that increasing growth rate will monotonically increase the size of daughter cells in subsequent generations until they reach their final size, irrespective of the time at which growth rate is increased (lower panels), with some minor deviations.
(EPS)

S4 Fig Correlation between *V*_*dau*_ and *T*_*G*1_ for a range of mutants. Data from [52], after filtering out mutants which displayed changes in growth rate (as described in [52]).
(EPS)

S5 Fig Mode-locking of the Pfeuty model to periodic forcing. (A) shows the predicted change in cell cycle phase in response to a 2.15 minute, 10% increase in the parameter *s*_*x*,2_ (denoted Δ*ϕ*(*t*), red line). This perturbation is repeatedly applied at a period 5 minutes less than the unforced cell cycle period (dashed line). The predicted phase of entrainment is given by the point of intersection of these lines where Δ*ϕ*′(*t*)<0 (red circle). (B) shows the simulated results evaluating the prediction made in (A). The shaded area represents the time at which the perturbation is applied. The dashed vertical line represents the prediction made in (A). The stability of the mode-locking is demonstrated by the consistent phase relationship between the perturbation and the timing of cell division.
(EPS)

S6 Fig Linear fit of *V*_*dau*_ and *T*_*G*1_ at constant growth rate and different glucose levels. Data from [46]. This quantifies the negative correlation between *V*_*dau*_ and *T*_*G*1_ observed as glucose levels change.
(EPS)

S7 Fig Examples of how different parameter combinations can produce the same eventual change in behaviour, but with different dynamic responses. Responses to changes in two pairs of parameters are analysed: *k*_*dcmp*_ and *k*_*smbM*_ (A, B, C); and *k*_*dcm*_ and *k*_*sn*3_ (D, E, F). In each case, the different responses of the individual parameters are combined to give the same eventual change in *V*_*div*_ and *T*_*div*_ (A, D). However, each case has a distinct dynamic response (compare B,C to E,F).
(EPS)
