# Supplementary material for: Model-Based Analysis of Cell Cycle Responses to Dynamically Changing Environments
Source: PLoS Comput Biol. 2016 Jan 7;12(1):e1004604. doi: 10.1371/journal.pcbi.1004604 (PMC4704810; doi:10.1371/journal.pcbi.1004604)
Supplement: S1 Text — (PDF) [file pcbi.1004604.s001.pdf]

Supplementary Information

Model-based analysis of cell cycle responses to dynamically changing environments

DD Seaton, J Krishnan

Contents

1 Supplementary Figures **2**

2 Model equations **9**

3 Model parameters of particular interest **17**

4 Sensitivity analysis **18**

4.1 Computing sensitivities 18

4.2 Characterising monotonic and nonmonotonic sensitivity responses 19

5 Combining parameter perturbations to produce the same change in macroscopic behaviour **19**

6 Analysis of phenomenological model of the cell cycle under perturbations **21**

6.1 Relationship between the durations of the G1 and S/G2/M phases 21

6.2 Phase responses and fraction of mass donated to daughter cells 22

6.3 Inferring the correspondence between cell cycle characteristics of populations and single cells 24

1 Supplementary Figures

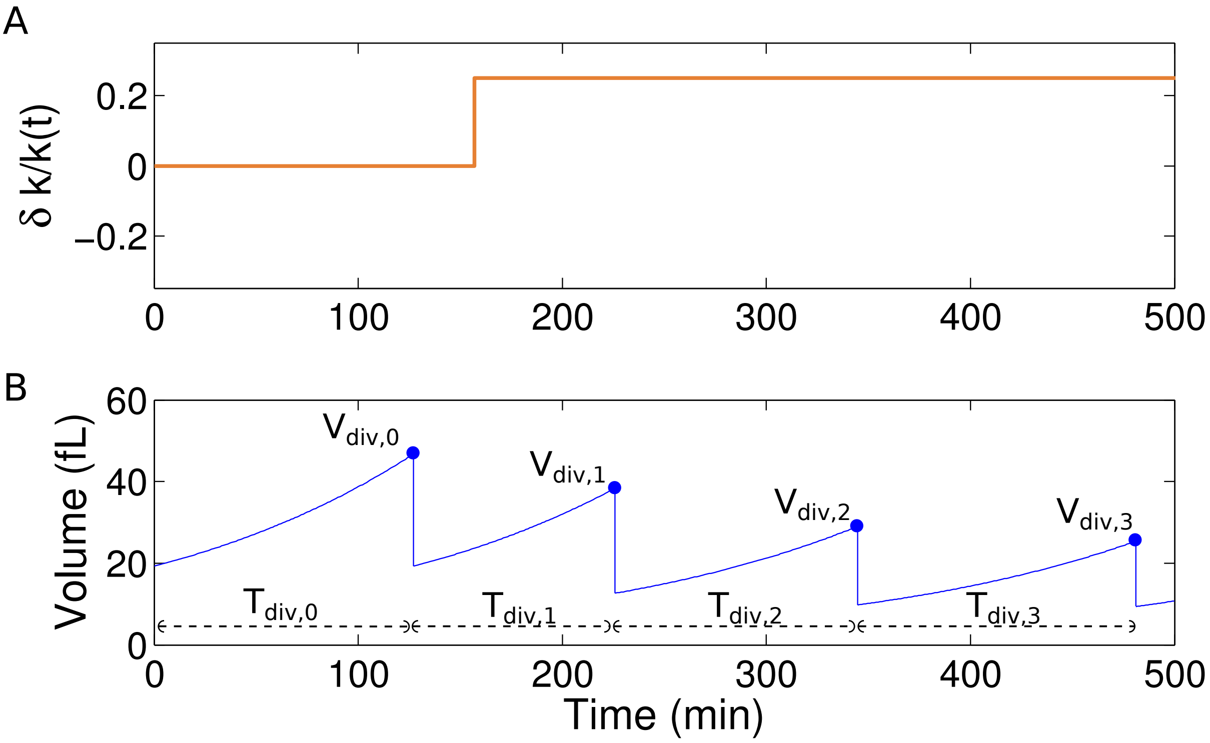

Figure S1: Simulating the dynamic response of the cell cycle of a step-change in parameters. The parameter $k_{s,bS}$ in the Barik model undergoes a step-change (A), which changes the volume at division (V_{div}) and cell cycle duration (T_{div}) in subsequent generations (B).

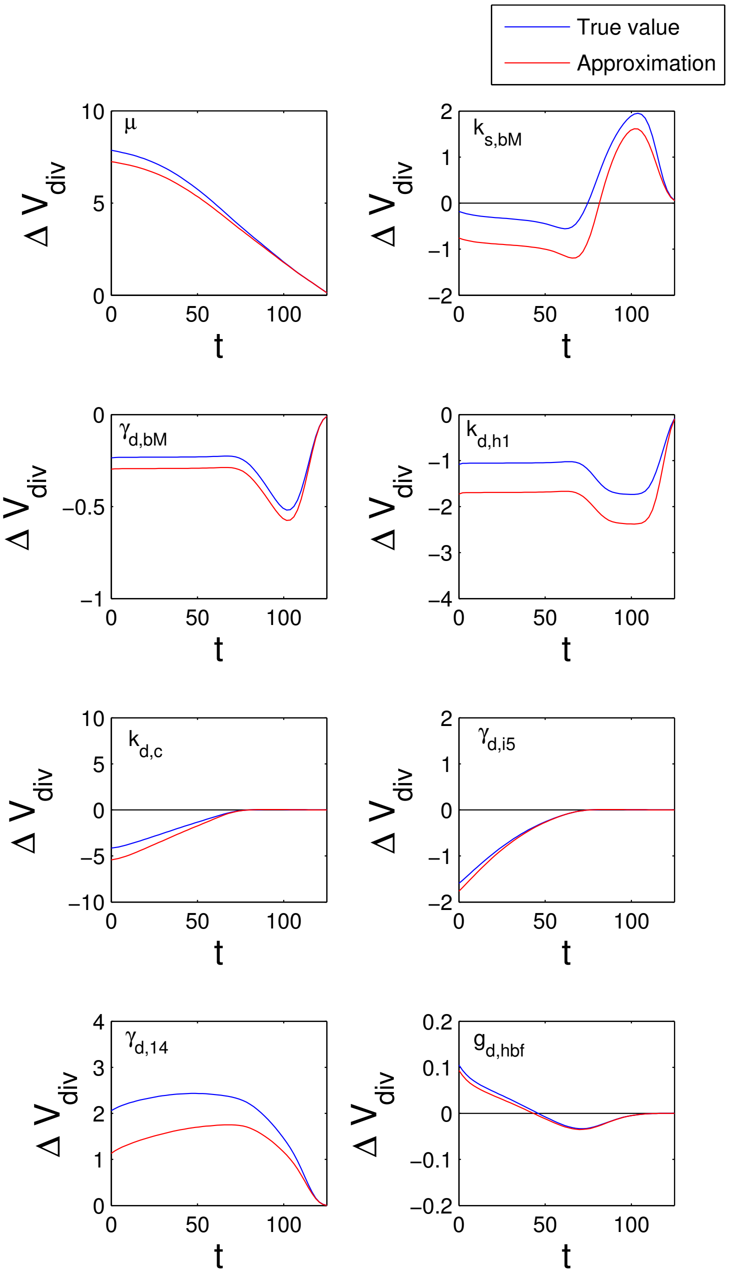

Figure S2: Qualitative assessment of approximation of model behaviour by extrapolation of local sensitivity in the case of the Barik model. Eight different parameters were increased by 20% at different times during the simulation, with the V_{div} in the first generation compared to an estimate based on the sensitivity analysis.

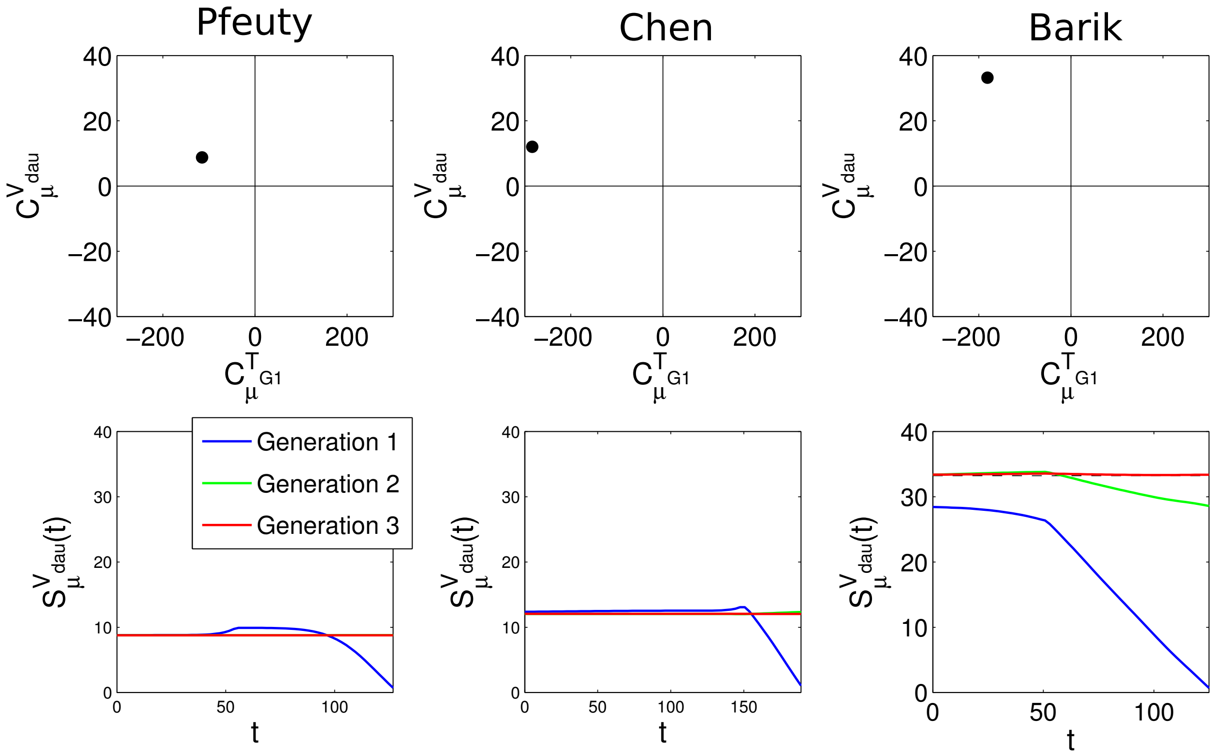

Figure S3: Model sensitivity to changes in the growth rate parameter, μ. In all three models, increasing growth rate leads to larger daughter cells and a reduced duration of G1 (upper panels), consistent with experimental observations. In addition, the models consistently predict that increasing growth rate will monotonically increase the size of daughter cells in subsequent generations until they reach their final size, irrespective of the time at which growth rate is increased (lower panels), with some minor deviations.

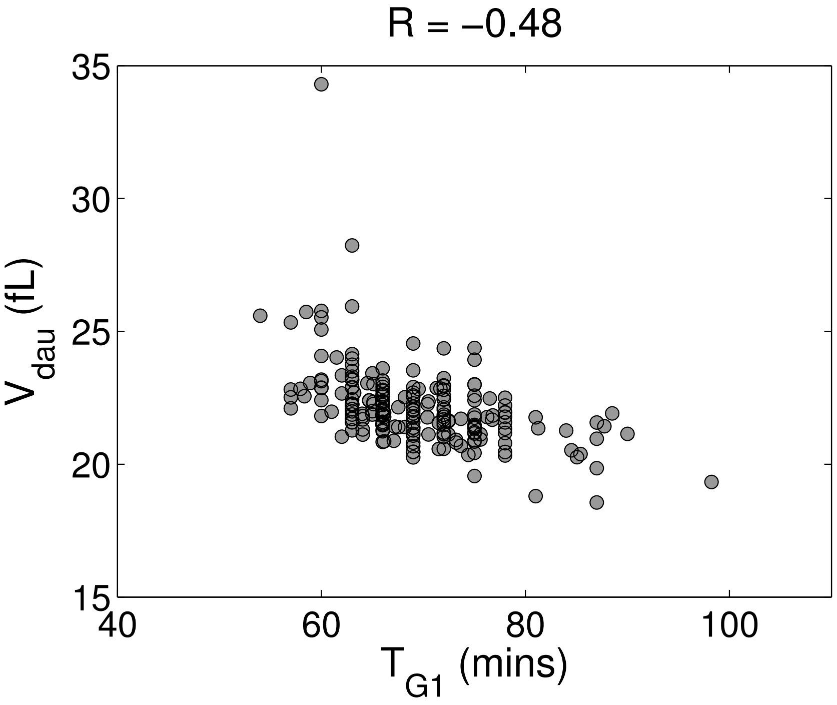

Figure S4: Correlation between V_{dau} and T_{G1} for a range of mutants. Data from [1], after filtering out mutants which displayed changes in growth rate (as described in [1]).

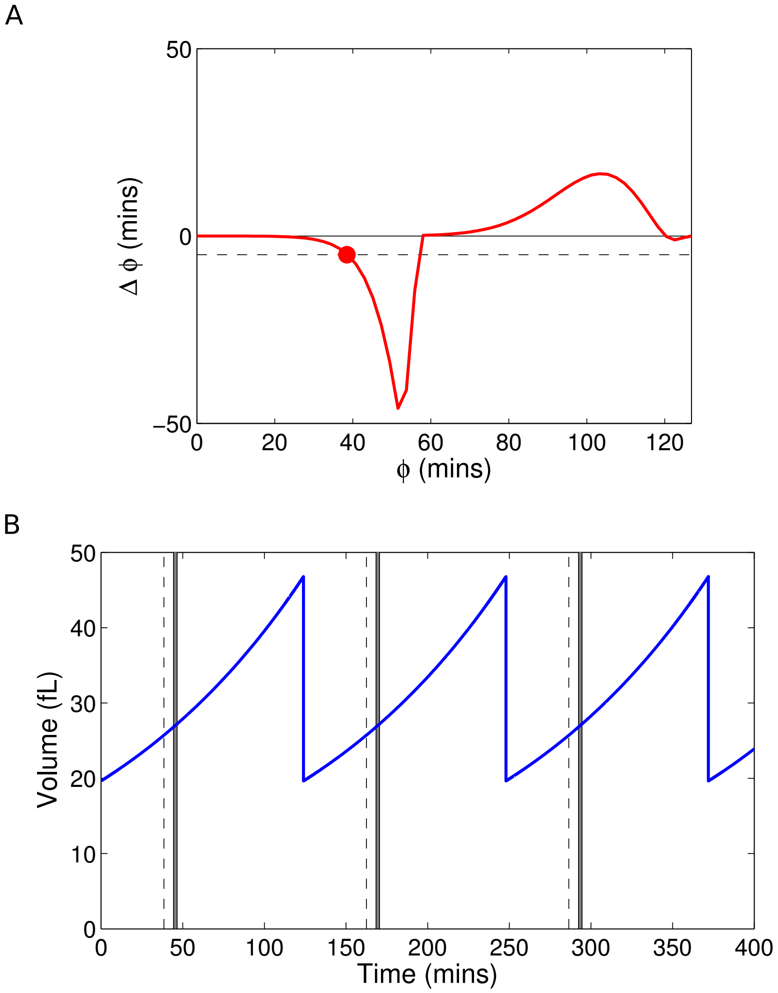

Figure S5: Mode-locking of the Pfeuty model to periodic forcing. (A) shows the predicted change in cell cycle phase in response to a 2.15 minute, 10% increase in the parameter $s_{x,2}$ (denoted $\Delta\phi(t)$, red line). This perturbation is repeatedly applied at a period 5 minutes less than the unforced cell cycle period (dashed line). The predicted phase of entrainment is given by the point of intersection of these lines where $\Delta\phi'(t) < 0$ (red circle). (B) shows the simulated results evaluating the prediction made in (A). The shaded area represents the time at which the perturbation is applied. The dashed vertical line represents the prediction made in (A). The stability of the mode-locking is demonstrated by the consistent phase relationship between the perturbation and the timing of cell division.

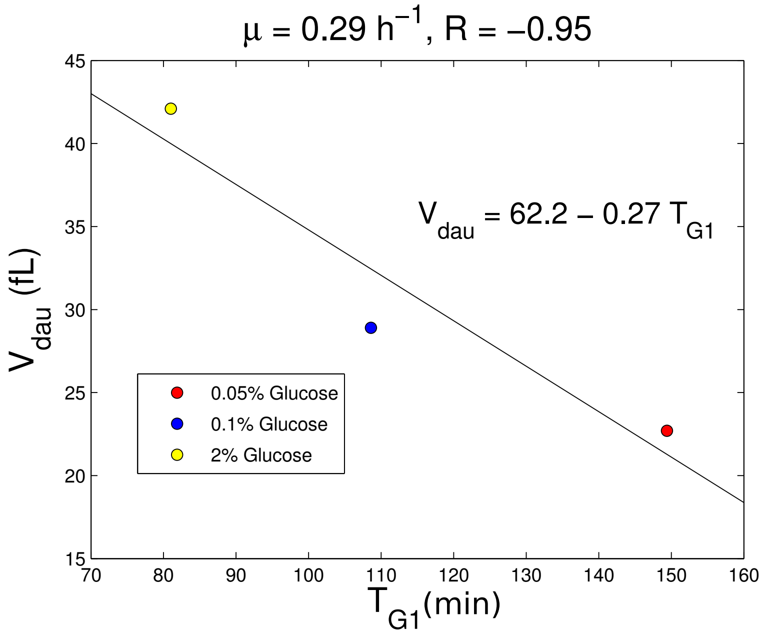

Figure S6: Linear fit of V_{dau} and T_{G1} at constant growth rate and different glucose levels. Data from [2]. This quantifies the negative correlation between V_{dau} and T_{G1} observed as glucose levels change.

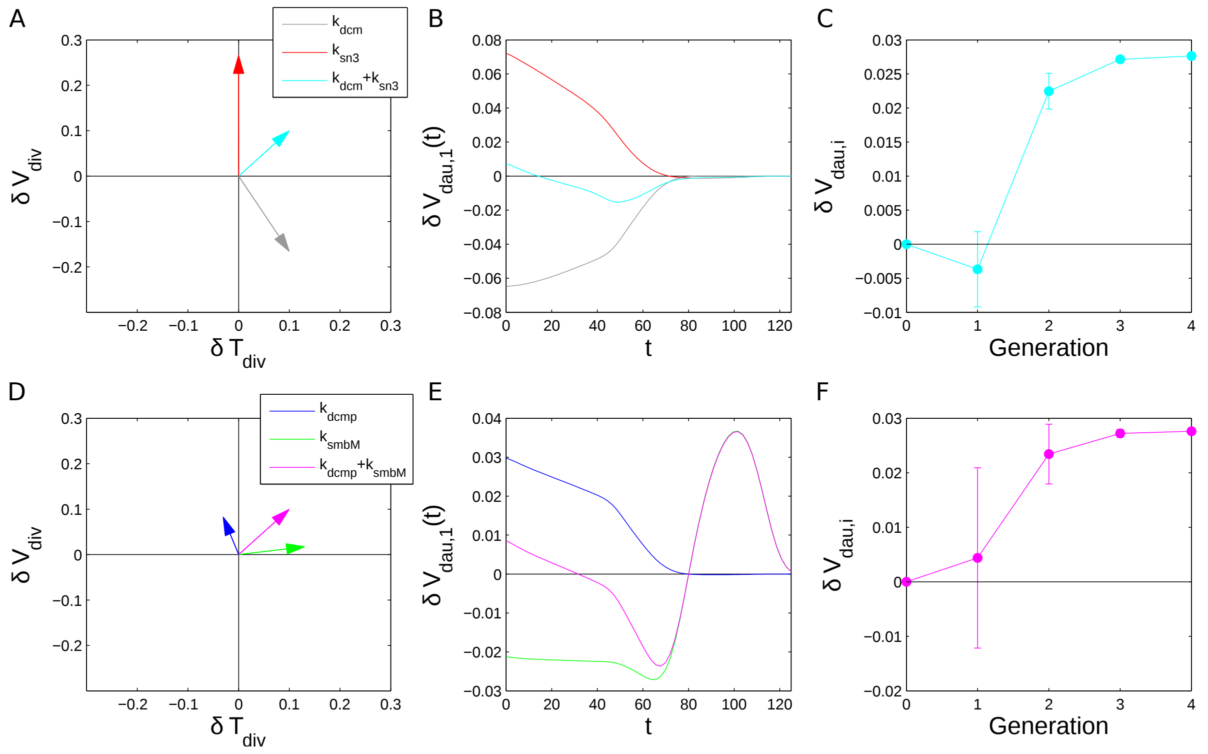

Figure S7: Examples of how different parameter combinations can produce the same eventual change in behaviour, but with different dynamic responses. Responses to changes in two pairs of parameters are analysed: k_{dcmp} and k_{smbM} (A, B, C); and k_{dcm} and k_{sn3} (D, E, F). In each case, the different responses of the individual parameters are combined to give the same eventual change in V_{div} and T_{div} (A, D). However, each case has a distinct dynamic response (compare B,C to E,F).

2 Model equations

All three models share the basic pattern of growth, budding and division, with cell size (V) growing exponentially, as given by:

$$\frac{dV}{dt} = \mu V \tag{1}$$

The link between growth in cell volume and and the macroscopic observables characterising the cell cycle is then given by (as in the Main Text):

$$\begin{aligned} V_{div} &= V_{dau} e^{\mu T_{div}} \\ V_{bud} &= V_{dau} e^{\mu T_{bud}} \\ f &= V_{dau}/V_{div} \end{aligned} \tag{2}$$

This description is coupled to models of the molecular mechanisms of cell cycle progression through thresholds in the concentrations of key components. In each model, a component controls the initiation of S-phase and budding, and this event occurs when the concentration of this component increases through a given threshold. In the case of the Barik model, this threshold is as specified in the original paper [3], while for the Pfeuty and Chen models a threshold was chosen such that the daughter cell volume is an appropriate fraction of the mother cell volume. Similarly, each model includes a component controls the initiation of cytokinesis, and this event occurs when the concentration of this component decreases through a given threshold. All three models already specify such a threshold, and we use these as specified in the original papers. All three models were run with the same basal growth rate ($\mu = 0.007\ min^{-1}$). Parameters determining how cell size (V) modulates cell cycle dynamics were rescaled in the Pfeuty and Chen models to make V_{div} the same for all

three models (and therefore in common units, of fL), with details described below.

Pfeuty model

The Pfeuty model was described in [4], and provides a minimal pseud-biochemical description of cell cycle dynamics. The model equations are:

$$\begin{aligned} \frac{d[X]}{dt} &= s_{x1} + s_{x2}V - (d_x + c_{xy}[Y])[X] + a_x[X]^2 \\ \frac{d[Y]}{dt} &= s_y[X]^2 - d_y[Y] \end{aligned} \tag{3}$$

In this model, budding occurs when $[X]$ increases above 0.2. Division occurs when $[Y]$ decreases below 2.

All parameters were rescaled by dividing by a factor of 2.86 to maintain the dynamics of the model at the common growth rate ($\mu = 0.007\ min^{-1}$). We note that this does not change the behaviour of the model, but merely amounts to a rescaling of the time coordinate. The parameter $s_{x,2}$ was additionally rescaled by dividing by a factor of 20 to put cell size in common units with the Barik model.

Chen model

The Chen model was originally described in [5]. A modified version of this was analysed in [6], and it is this version that we use here. The model equations are:

$$\begin{aligned} \frac{d[Cln2]}{dt} &= V\left(k'_{s,n2} + k''_{s,n2}[SBF]\right) - k_{d,n2}[Cln2] \\ \frac{d[Clb2]_T}{dt} &= V\left(k'_{s,b2} + k''_{s,b2}[Mcm1]\right) - \left(k'_{d,b2} + \left(k''_{d,b2} - k'_{d,b2}\right)[Hct1]\right. \\ &\quad \left. + k'''_{d,b2}[Cdc20]\right)[Clb2]_T \end{aligned}$$

$$
\begin{aligned}
\frac{d[Hct1]}{dt} &= \frac{\left(k'_{a,t1} + k''_{a,t1}[Cdc20]\right)(1-[Hct1])}{J_{a,t1}+1-[Hct1]} - \frac{V_{i,t1}[Hct1]}{[J_{i,t1}+[Hct1]]} \\
\frac{d[Cdc20]_T}{dt} &= \left(k'_{s,20} + k''_{s,20}[Clb2]\right) - k_{d,20}[Cdc20]_T \\
\frac{d[Cdc20]}{dt} &= k_{a,20}\left([Cdc20]_T - [Cdc20]\right) - \left(V_{i,20} - k_{d,20}\right)[Cdc20] \\
\frac{d[Clb5]_T}{dt} &= V\left(k'_{s,b5} + k''_{s,b5}[MBF]\right) - \left(k'_{d,b5} + k''_{d,b5}[Cdc20]\right)[Clb5]_T
\end{aligned}
$$

$$
\begin{aligned}
\frac{d[Sic1]_T}{dt} &= k'_{s,c1} + k''_{s,c1}[Swi5] - \left(k_{d1,c1} + \frac{V_{d2c1}}{J_{d2,c1}+[Sic1]_T}\right)[Sic1]_T \\
\frac{d[Clb5|Sic1]}{dt} &= k_{as,b5}[Clb5][Sic1] - \left(k_{di,b5} + k'_{d,b5} + k''_{d,b5}[Cdc20] + k_{d1,c1}\right. \\
&\quad \left. + \frac{V_{d2c1}}{J_{d2,c1}+[Sic1]_T}\right)[Clb5|Sic1] \\
\frac{d[Clb2|Sic1]}{dt} &= k_{as,b2}[Clb2][Sic1] - \left(k_{di,b2} + k'_{d,b2} + \left(k''_{d,b2} - k'_{d,b2}\right)[Hct1]\right. \\
&\quad \left. + k'''_{d,b2}[Cdc20] + k_{d1,c1} + \frac{V_{d2c1}}{J_{d2,c1}+[Sic1]_T}\right)[Clb2|Sic1] \\
V_{d2,c1} &= k_{d2,c1}\left(\epsilon_{c1,n3}[Cln3]^* + \epsilon_{c1,k2}[Bck2] + [Cln2] + \epsilon_{c1,b5}[Clb5] + \epsilon_{c1,b2}[Clb2]\right) \\
[Mcm1] &= G\left(k_{a,mcm}[Clb2], k_{i,mcm}, J_{a,mcm}, J_{i,mcm}\right) \\
[Swi5] &= G\left(k_{a,swi}[Cdc20], k'_{i,swi} + k''_{i,swi}[Clb2], J_{a,swi}, J_{i,swi}\right) \\
[SBF] &= [MBF] = G\left(V_{a,sbf}, k'_{i,sbf} + k''_{i,sbf}[Clb2], J_{a,sbf}, J_{i,sbf}\right) \\
V_{a,sbf} &= k_{a,sbf}\left([Cln2] + \epsilon_{sbf,n3}\left([Cln3]^* + [Bck2]\right) + \epsilon_{sbf,b5}[Clb5]\right) \\
V_{i,t1} &= k'_{i,t1} + k''_{i,t1}\left([Cln3]^* + \epsilon_{i,t1,n2}[Cln2] + \epsilon_{i,t1,b5}[Clb5] + \epsilon_{i,t1,b2}[Clb2]\right) \\
[Clb2]_T &= [Clb2] + [Clb2|Sic1] \\
[Clb5]_T &= [Clb5] + [Clb5|Sic1] \\
[Sic1]_T &= [Sic1] + [Clb2|Sic1] + [Clb5|Sic1] \\
[Bck2] &= V[Bck2]^0 \\
[Cln3]^* &= [Cln3]_{max}\frac{VD_{n3}}{J_{n3}+VD_{n3}}
\end{aligned} \tag{4}
$$

Goldbeter Koshland function:

$$G(a,b,c,d) = \frac{2ad}{b-a+bc+ad+\sqrt{(b-a+bc+ad)^2-4ad(b-a)}} \tag{5}$$

In this model, budding occurs when $[Cln2]$ increases above 0.03. Division occurs when $[Clb2]_T$ decreases below 0.05.

The parameters $k^{'}_{s,n2}$, $k^{''}_{s,n2}$, $k^{'}_{s,b2}$, $k^{''}_{s,b2}$, $k^{'}_{s,b5}$, $k^{''}_{s,b5}$, $[Bck]^0$, and D_{n3} were rescaled by dividing by a factor of 33.3 to put cell size in common units with the Barik model.

Barik model

The Barik model was described in [3], and provides a detailed description of cell cycle dynamics in the form of mass-action kinetics. The model equations are:

$$
\begin{aligned}
\frac{d[Cln3]}{dt} &= k_{s,n3}\left(\frac{V^2}{P^2}\right)[Mn3] - \gamma_{n3}[Cln3] \\
\frac{d[ClbM]}{dt} &= k_{s,bM}\left(\frac{V}{P}\right)[MbM] - \frac{k_{d,bMa}}{V}[Cdh1][ClbM] - \frac{k_{d,bMi}}{V}[Cdh1P_T][ClbM] - \gamma_{bM}[ClbM] \\
\frac{d[ClbS]}{dt} &= k_{s,bS}\left(\frac{V}{P}\right)[MbS]\gamma_{bS}[ClbS] \\
\frac{d[Cdc14]}{dt} &= k_{s,c14}\left(\frac{V}{P}\right) + (k_{d,r} + \gamma_{t1})[RENT_T] - \frac{k_{a,r}}{V}[Net1R_T][Cdc14]\gamma_{c14}[Cdc14] \\
\frac{d[SBF]}{dt} &= k_{s,bf}V + (k_{d,c} + \gamma_{i5})([Cmp] + [CmpP_1] + [CmpP_2]) \\
&+ \left(\frac{k_{d,bf}}{V}[Hbf] + \frac{k'_{d,bf}}{V}[Cdc14]\right)[SBFP_1] \\
&- \left(\frac{k_{a,c}}{V}([Whi5] + [Whi5P_1] + [Whi5P_2]) + \frac{k_{p,bf}}{V}[ClbM] + \gamma_{bf}\right)[SBF] \\
\frac{d[SBFP_i]}{dt} &= \frac{k_{p,bf}}{V}[ClbM][SBFP_{i-1}] + \left(\frac{k_{d,bf}}{V}[Hbf] + \frac{k'_{d,bf}}{V}[Cdc14]\right)[SBFP_{i+1}] \\
&- \left(\frac{k_{p,bf}}{V}[ClbM] + \frac{k_{d,bf}}{V}[Hbf] + \frac{k'_{d,bf}}{V}[Cdc14] + \gamma_{bf}\right)[SBFP_i]; 1 \leq i \leq 3 \\
\frac{d[SBFP_4]}{dt} &= \frac{k_{p,bf}}{V}[ClbM][SBFP_3] - \left(\frac{k_{d,bf}}{V}[Hbf] + \frac{k'_{d,bf}}{V}[Cdc14] + \gamma_{bf}\right)[SBFP_4] \\
\frac{d[Hbf]}{dt} &= k_{s,hbf}\left(\frac{V}{P}\right)[Mhbf] - \gamma_{hbf}[Hbf] \\
\frac{d[Whi5]}{dt} &= k_{s,i5}\left(\frac{V}{P}\right)[Mi5] + (k_{d,c} + \gamma_{b,f})[Cmp] + \left(\frac{k_{d,i5}}{V}[Hi5] + \frac{k'_{d,i5}}{V}[Cdc14]\right)[Whi5P_1] \\
&- \left(\frac{k_{a,c}}{V}[SBF] + \frac{k_{p,i5}}{V}[Cln3] + \frac{k'_{p,i5}}{V}[ClbS] + \gamma_{i5}\right)[Whi5]
\end{aligned}
$$

$$\frac{d[Whi5P_i]}{dt} = (k_{d,c} + \gamma_{b,f})[CmpP_i] + \left(\frac{k_{p,i5}}{V}[Cln3] + \frac{k'_{p,i5}}{V}[ClbS]\right)[Whi5P_{i-1}]$$

$$+ \left(\frac{k_{d,i5}}{V}[Hi5] + \frac{k'_{d,i5}}{V}[Cdc14]\right)[Whi5P_{i+1}]$$

$$- \left(\frac{k_{a,c}}{V}[SBF] + \frac{k_{p,i5}}{V}[Cln3] + \frac{k'_{p,i5}}{V}[ClbS] + \frac{k_{d,i5}}{V}[Hi5] + \frac{k'_{d,i5}}{V}[Cdc14] + \gamma_{i5}\right)[Whi5P_i]$$

$$1 \le i \le 2$$

$$\frac{d[Whi5P_i]}{dt} = \left(\frac{k_{p,i5}}{V}[Cln3] + \frac{k'_{p,i5}}{V}[ClbS]\right)[Whi5P_{i-1}] + \left(\frac{k_{d,i5}}{V}[Hi5] + \frac{k'_{d,i5}}{V}[Cdc14]\right)[Whi5P_{i+1}]$$

$$- \left(\frac{k_{a,c}}{V}[SBF] + \frac{k_{p,i5}}{V}[Cln3] + \frac{k'_{p,i5}}{V}[ClbS] + \frac{k_{d,i5}}{V}[Hi5] + \frac{k'_{d,i5}}{V}[Cdc14] + \gamma_{i5}\right)[Whi5P_i]$$

$$3 \le i \le 5$$

$$\frac{d[Whi5P_6]}{dt} = \left(\frac{k_{p,i5}}{V}[Cln3] + \frac{k'_{p,i5}}{V}[ClbS]\right)[Whi5P_5] - \left(\frac{k_{d,i5}}{V}[Hi5] + \frac{k'_{d,i5}}{V}[Cdc14] + \gamma_{i5}\right)[Whi5P_6]$$

$$\frac{d[Hi5]}{dt} = k_{s,hi5}\left(\frac{V}{P}\right)[Mhi5] - \gamma_{hi5}[Hi5]$$

$$\frac{d[Net1]}{dt} = k_{s,t1}\left(\frac{V}{P}\right)[Mt1] + (k_{d,r} + \gamma_{c14})[RENT] + \frac{k_{d,t1}V}{[}Ht1][NetP_1]$$

$$- \left(\frac{k_{a,r}}{V}[Cdc14] + \frac{k_{p,t1}}{V}[ClbM] + \gamma_{t1}\right)[Net1]$$

$$\frac{d[Net1P_i]}{dt} = (k_{d,r} + \gamma_{c14})[RENTP_i] + \frac{k_{p,t1}}{V}[ClbM][Net1P_{i-1}] + \frac{k_{d,t1}}{V}[Ht1][NetP_{i+1}]$$

$$- \left(\frac{k_{a,r}}{V}[Cdc14] + \frac{k_{p,t1}}{V}[ClbM] + \frac{k_{d,t1}}{V}[Ht1] + \gamma_{t1}\right)[Net1P_i]; 2 \le i \le 5$$

$$\frac{d[Net1P_i]}{dt} = \frac{k_{p,t1}}{V}[ClbM][Net1P_{i-1}] + \frac{k_{d,t1}}{V}[Ht1][NetP_{i+1}]$$

$$- \left(\frac{k_{p,t1}}{V}[ClbM] + \frac{k_{d,t1}}{V}[Ht1] + \gamma_{t1}\right)[Net1P_i]; 6 \le i \le 7$$

$$\frac{d[Net1P_8]}{dt} = \frac{k_{p,t1}}{V}[ClbM][Net1P_7] - \left(\frac{k_{d,t1}}{V}[Ht1] + \gamma_{t1}\right)[Net1P_8]$$

$$\frac{d[RENT]}{dt} = \frac{k_{a,r}}{V}[Cdc14][Net1] + \frac{k_{d,nt}}{V}[Ht1][RENTP_1]$$

$$- \left(k_{d,r} + \frac{k_{p,nt}}{V}[ClbM] + \gamma_{t1} + \gamma_{c14}\right)[RENT]$$

$$\frac{d[RENTP_i]}{dt} = \frac{k_{a,r}}{V}[Cdc14][Net1P_i] + \frac{k_{p,nt}}{V}[ClbM][RENTP_{i-1}] + \frac{k_{d,nt}}{V}[Ht1][RENTP_{i+1}] - \left(k_{d,r} + \frac{k_{p,nt}}{V}[ClbM] + \frac{k_{d,nt}}{V}[Ht1] + \gamma_{t1} + \gamma_{c14}\right)[RENTP_i]; 2 \leq i \leq 4$$

$$\frac{d[RENTP_5]}{dt} = \frac{k_{a,r}}{V}[Cdc14][Net1P_5] + \frac{k_{p,nt}}{V}[ClbM][RENTP_4] - \left(k_{d,r} + \frac{k_{d,nt}}{V}[Ht1] + \gamma_{t1} + \gamma_{c14}\right)[RENTP_5]$$

$$\frac{d[Ht1]}{dt} = k_{s,ht1}\left(\frac{V}{P}\right)[Mht1] - \gamma_{ht1}[Ht1]$$

$$\frac{d[Cmp]}{dt} = \left(\frac{k_{a,c}}{V}\right)[SBF][Whi5] + \left(\frac{k_{d,cm}}{V}[Hi5] + \frac{k'_{d,cm}}{V}[Cdc14]\right)[CmpP_1] - \left(k_{d,c} + \frac{k_{p,cm}}{V}[Cln3] + \frac{k'_{p,cm}}{V}[ClbS] + \gamma_{b,f} + \gamma_{i5}\right)[Cmp]$$

$$\frac{d[CmpP_1]}{dt} = \left(\frac{k_{a,c}}{V}\right)[SBF][Whi5P_1] + \left(\frac{k_{p,cm}}{V}[Cln3] + \frac{k'_{p,cm}}{V}[ClbS]\right)[Cmp] + \left(\frac{k_{d,cm}}{V}[Hi5] + \frac{k'_{d,cm}}{V}[Cdc14]\right)[CmpP_2] - \left(k_{d,c} + \frac{k_{d,cm}}{V}[Hi5] + \frac{k'_{d,cm}}{V}[Cdc14] + \frac{k_{p,cm}}{V}[Cln3] + \frac{k'_{p,cm}}{V}[ClbS] + \gamma_{b,f} + \gamma_{i5}\right)[CmpP_1]$$

$$\frac{d[CmpP_2]}{dt} = \left(\frac{k_{a,c}}{V}\right)[SBF][Whi5P_2] + \left(\frac{k_{p,cm}}{V}[Cln3] + \frac{k'_{p,cm}}{V}[ClbS]\right)[CmpP_1] - \left(k_{d,c} + \frac{k_{d,cm}}{V}[Hi5] + \frac{k'_{d,cm}}{V}[Cdc14] + \gamma_{b,f} + \gamma_{i5}\right)[CmpP_2]$$

$$\frac{d[Cdh1]}{dt} = k_{s,h1}\left(\frac{V}{P}\right)[Mh1] + \left(\frac{k_{d,h1}}{V}[Cdc14] + \frac{k'_{d,h1}}{V}\right)[Cdh1P_1] - \left(\frac{k_{p,h1}}{V}[ClbS] + \frac{k'_{p,h1}}{V}[ClbM] + \gamma_{h1}\right)[Cdh1]$$

$$
\begin{aligned}
\frac{d[Cdh1P_i]}{dt} &= \left(\frac{k_{p,h1}}{V}[ClbS] + \frac{k'_{p,h1}}{V}[ClbM]\right)[Cdh1P_{i-1}] + \left(\frac{k_{d,h1}}{V}[Cdc14] + \frac{k'_{d,h1}}{V}\right)[Cdh1P_{i+1}] \\
&\quad - \left(\frac{k_{p,h1}}{V}[ClbS] + \frac{k'_{p,h1}}{V}[ClbM] + \frac{k_{d,h1}}{V}[Cdc14] + \frac{k'_{d,h1}}{V} + \gamma_{h1}\right)[Cdh1P_i]; 2 \le i \le 9 \\
\frac{d[Cdh1P_{10}]}{dt} &= \left(\frac{k_{p,h1}}{V}[ClbS] + \frac{k'_{p,h1}}{V}[ClbM]\right)[Cdh1P_9] - \left(\frac{k_{d,h1}}{V}[Cdc14] + \frac{k'_{d,h1}}{V} + \gamma_{h1}\right)[Cdh1P_{10}] \\
\frac{d[Mn3]}{dt} &= k_{s,mn3}P - \gamma_{mn3}[Mn3] \\
\frac{d[MbM]}{dt} &= k_{s,mbM}P - \gamma_{mbM}[MbM] \\
\frac{d[G_a]}{dt} &= \frac{k_{a,g}}{V}[SBF]\left(P - [G_a]\right) - k_{d,g}[G_a] \\
\frac{d[MbS]}{dt} &= k_{s,mbS}P - \gamma_{mbS}[MbS] \\
\frac{d[Mc14]}{dt} &= k_{s,mc14}P - \gamma_{mc14}[Mc14] \\
\frac{d[Mhbf]}{dt} &= k_{s,mhbf}P - \gamma_{mhbf}[Mhbf] \\
\frac{d[Mi5]}{dt} &= k_{s,mi5}P - \gamma_{mi5}[Mi5] \\
\frac{d[Mhi5]}{dt} &= k_{s,mhi5}P - \gamma_{mhi5}[Mhi5] \\
\frac{d[Mt1]}{dt} &= k_{s,mt1}P - \gamma_{mt1}[Mt1] \\
\frac{d[Mht1]}{dt} &= k_{s,mht1}P - \gamma_{mht1}[Mht1] \\
\frac{d[Mh1]}{dt} &= k_{s,mh1}P - \gamma_{mh1}[Mh1] \\
[Net1R_T] &= \sum_{i=0}^{5}[Net1P_i] \\
[RENT_T] &= \sum_{i=0}^{5}[RENTP_i] \\
[Cdh1P_T] &= \sum_{i=1}^{10}[Cdh1P_i]
\end{aligned}
$$

In this model, budding occurs when $[ClbS]$ increases above 37.5nM. Division occurs when $[ClbM]$ decreases below 12.5nM.

3 Model parameters of particular interest

While there is not, in general, a straightforward mapping between parameters in different models, there are sets of analogous parameters that represent similar molecular mechanisms. These include parameters involved in cyclin synthesis and degradation (through the APC), as discussed in the Main Text. Here, we detail which sets of parameters we consider to represent these processes in each model.

Cln3 synthesis

The synthesis of the G1 cyclin Cln3 is represented in both the Chen and Barik models, and is represented by the parameters D_{n3} and $k_{s,n3}$, respectively. The component X in the Pfeuty model plays the role of initiating Start in this model, and its rate of synthesis is controlled by the parameter $s_{x,2}$.

Mitotic cyclin synthesis

The synthesis of mitotic cyclin is represented in both the Chen and Barik models, and is represented by the parameters $k'_{s,b2}$ and $k_{s,bM}$, respectively.

APC synthesis

The APC subunit Cdc20 is represented in the Chen model, and its synthesis is represented by the parameter $k'_{s,20}$. The APC subunit Cdh1 is represented in the Barik model, and its synthesis is represented by the parameter $k_{s,h1}$.

4 Sensitivity analysis

4.1 Computing sensitivities

In order to perform sensitivity analysis for a sustained change in parameters, the models are first simulated from given initial conditions over multiple cycles until the state of the cell at the beginning of the cell cycle had converged. At this point, the parameter is changed in a step-wise fashion and the simulation continued, again until convergence of the state at the beginning of one cycle. For the step change, the parameter under consideration was multiplied by a factor of 1.001, corresponding to a 0.1% change in the parameter. This was found to be a large enough change to make the sensitivity measurements unaffected by numerical errors in the ODE solver, but small enough to give an accurate measure of the first-derivative of the observables to changes in the parameter. The solver used was ode15s, in MATLAB, run with absolute and relative tolerances of 10^{-10}. Repeating the analysis with tolerances of 10^{-13} had no significant effect on the results.

Having simulated cell cycle behaviour with the basal and perturbed sets of parameters, it is possible to calculate the sensitivity of the observables to changes in the parameter in question according to:

$$C_{k_i}^{Q} \approx k_i \frac{\Delta Q}{\Delta k_i} \tag{6}$$

Where $\Delta Q = Q_{perturbed} - Q_{basal}$ and $\Delta k_i / k_i = 0.001$. For a given parameter perturbation, this sensitivity gives a first-order estimate of the change in behaviour:

$$\Delta Q_{1^{st}} = \frac{\Delta k_i}{k_i} . C_{k_i}^{Q} \tag{7}$$

An analogous calculation was performed for calculating the dynamic sensitivities, $S_k^{Q_i}$, by performing step changes in parameters at different times during the cell cycle and tracking changes in behaviour down subsequent generations. An illustration of a similar simulation

(though with larger changes in parameters) is shown in Supplementary Figure S1.

4.2 Characterising monotonic and nonmonotonic sensitivity responses

In the Main Text, the pervasiveness of nonmonotonic changes in T_{G1} down generations in response to a step-change in parameters is discussed (Figure 4). For a perturbation of a parameter k at time t, the sensitivity of T_{G1} down generations is characterised by the sequence:

$$S_k^{T_{G1,1}}(t), S_k^{T_{G1,2}}(t), ..., S_k^{T_{G1,n}}(t) \tag{8}$$

In order to quantify montonicity, the sequence of $S_k^{T_{G1,i}}(t), i = 1, 2, 3$ was evaluated for monotonicity for each of the 60 timepoints for which sensitivity functions were computed. The presented fractions of monotonic and nonmonotonic responses are thus the fraction of time for which the function $S_k^{T_{G1,i}}(t)$ gives a monotonic or nonmonotonic response, respectively, averaged across all parameters.

5 Combining parameter perturbations to produce the same change in macroscopic behaviour

Different parameter perturbations may result in a wide range of different behaviours, both in dynamic response and under constant conditions. In this context, it is useful to be able to compare the dynamic responses of different parameter perturbations. This allows for assessment of whether a parameter perturbation produces an especially fast or slow response in cell cycle behaviour, for example. Since different parameter perturbations result in different eventual cell cycle behaviours, it is not clear how comparisons of the dynamics of different perturbations can be evaluated. In order to make such comparisons, it is possible to exploit the linearity in the neighbourhood of the basal parameter set to find parameter perturbations

which result in identical changes in the eventual macroscopic cell cycle behaviour. In particular, changes in behaviour are given by (following [7, 8]):

$$\begin{pmatrix} \delta V_{div} \\ \delta T_{div} \end{pmatrix} = \begin{pmatrix} C_{k_i}^{V_{div}} & C_{k_j}^{V_{div}} \\ C_{k_i}^{T_{div}} & C_{k_j}^{T_{div}} \end{pmatrix} \begin{pmatrix} \delta k_i/k_i \\ \delta k_j/k_j \end{pmatrix} \tag{9}$$

Therefore, for two parameters with linearly independent sensitivity vectors $(C_k^{V_{div}}, C_k^{T_{div}})^T$, the parameter perturbation required to obtain the eventual change in behaviour $(\delta V_{div}, \delta T_{div})^T$ is given by:

$$\delta\eta = \begin{pmatrix} \delta k_i/k_i \\ \delta k_j/k_j \end{pmatrix} = \begin{pmatrix} C_{k_i}^{V_{div}} & C_{k_j}^{V_{div}} \\ C_{k_i}^{T_{div}} & C_{k_j}^{T_{div}} \end{pmatrix}^{-1} \begin{pmatrix} \delta V_{div} \\ \delta T_{div} \end{pmatrix} \tag{10}$$

The significance of this is illustrated in Supplementary Figure S7.

From the preceding analysis, it is clear that the cell cycle models considered are capable of a multitude of different dynamic responses. One straightforward way of simplifying and comparing these results is to consider what the average response is to a particular perturbation, and how variable that response is. This is sensible in the context of perturbations of populations of cells, where the timing of an external perturbation relative to the cell cycle phase of a particular cell is essentially random. The average change in daughter cell size in generation i is denoted by $\delta\bar{V}_{dau,i}$ and given by:

$$\delta\bar{V}_{dau,i} = \frac{1}{T_{div}} \int_{t=0}^{T_{div}} \delta V_{dau,i}(t)dt \tag{11}$$

The distribution of daughter cell sizes around this average is characterised by the standard deviation, given by $\sigma_{V_{dau,i}}$:

$$\sigma_{V_{dau,i}} = \sqrt{\frac{1}{T_{div}} \int_{t=0}^{T_{div}} \left(\delta V_{dau,i}(t) - \delta\bar{V}_{dau,i}\right)^2 dt} \tag{12}$$

Supplementary Figures S7C,E can be compared to assess how these values differ for the

two example combinations of parameters considered here.

This analysis is useful for comparing the dynamics of cell cycle response to different parameter perturbations while avoiding artefacts resulting from differences in the eventual change in behaviour elicited by these perturbations. In the case of the glucose signalling case study (see Main Text), this allows assessment of the role of Net1 dephosphorylation in speeding up the response to changes in glucose levels (Figure 6).

6 Analysis of phenomenological model of the cell cycle under perturbations

6.1 Relationship between the durations of the G1 and S/G2/M phases

In this section, we use the phenomenological model of the cell cycle to derive a strict relationship between the sensitivities of the G1 and S/G2/M phases to perturbations (Equation 11 in the Main Text).

Under constant conditions, the volumes of a cell at budding and division are given by:

$$\begin{aligned} V_{div} &= V_{bud} e^{\mu T_{S/G2/M}} \\ V_{dau} &= V_{bud} e^{-\mu T_{G1}} \end{aligned} \tag{13}$$

These are related to the volume of the daughter cell at birth:

$$V_{div} - V_{bud} = V_{dau} \tag{14}$$

Substituting, we get:

$$\begin{aligned} V_{bud}e^{\mu T_{S/G2/M}} - V_{bud} &= V_{bud}e^{-\mu T_{G1}} \\ \implies e^{\mu T_{S/G2/M}} - 1 &= e^{-\mu T_{G1}} \end{aligned} \tag{15}$$

Differentiating with respect to a generic parameter, k, fixing $d\mu/dk = 0$, and noting $T_{G1} + T_{S/G2/M} = T_{div}$:

$$\frac{dT_{S/G2/M}}{dk} = -\frac{dT_{G1}}{dk}e^{-\mu T_{div}} \tag{16}$$

Noting that the fraction of mother cell volume donated to the daughter cell at division, f, is given by:

$$\begin{aligned} f &= \frac{V_{dau}}{V_{div}} \\ &= \frac{V_{dau}}{V_{dau}e^{\mu T_{div}}} \\ &= e^{-\mu T_{div}} \end{aligned} \tag{17}$$

we obtain:

$$\frac{dT_{S/G2/M}}{dk} = -f\frac{dT_{G1}}{dk} \tag{18}$$

6.2 Phase responses and fraction of mass donated to daughter cells

In this section, we use the phenomenological model to relate changes in cell cycle phase (i.e. changes in the timing of cell cycle events relative to a reference case) to changes in cell volume at budding and division at constant growth rate.

We consider a cell cycle which initially has a period T_0, a size at division $V_{div,0}$, and a daughter size $V_{dau,0}$, meaning the resulting fraction of volume given to the daughter

cell is $f_0 = V_{dau,0}/V_{div,0}$. Two perturbations are applied which result in changes in these characteristics down generations. The first set of characteristics are labelled $T_1, T_2, T_3, ...$, $V_{div,1}, V_{div,2}, V_{div,3}, ...$, $V_{dau,1}, V_{dau,2}, V_{dau,3}, ...$, giving daughter fractions $f_1, f_2, f_3, ...$, while the second are labelled $\tau_1, \tau_2, \tau_3, ...$, $\nu_{div,1}, \nu_{div,2}, \nu_{div,3}, ...$, and $\nu_{dau,1}, \nu_{dau,2}, \nu_{dau,3}, ...$, giving daughter fractions $\rho_1, \rho_2, \rho_3, ...$. Initially the cells are equally sized:

$$V_{dau,0} = \nu_{dau,0} \tag{19}$$

For subsequent generations, we have:

$$\begin{aligned} V_{dau,i} &= \frac{V_{dau,i+1} e^{-T_{i+1}\mu}}{f_{i+1}} \\ \nu_{dau,i} &= \frac{\nu_{dau,i+1} e^{-\tau_{i+1}\mu}}{\rho_{i+1}} \end{aligned} \tag{20}$$

Thus after n generations, using the equality from Equation 19:

$$\frac{V_{dau,n} \prod_{i=1}^{n} e^{-T_i\mu}}{\prod_{i=1}^{n} f_i} = \frac{\nu_{dau,n} \prod_{i=1}^{n} e^{-\tau_i\mu}}{\prod_{i=1}^{n} \rho_i} \tag{21}$$

Taking logarithms and rearranging:

$$\sum_{i=1}^{n} (\tau_i - T_i) = \frac{1}{\mu} \ln \left(\frac{\nu_{dau,n}}{V_{dau,n}} \prod_{i=1}^{n} \frac{f_i}{\rho_i} \right) \tag{22}$$

Since the perturbations applied are, by assumption, temporary, we can take the limit as $n \to \infty$, so that $V_{dau,n} \to \nu_{dau,n}$, and $\sum_{i=1}^{n} (\tau_i - T_i) \to \Delta\phi$, where $\Delta\phi$ is the phase difference, as defined in Equation 12 (Main Text). This gives:

$$\Delta\phi = \frac{1}{\mu} \ln \left(\prod_{i=1}^{\infty} \frac{f_i}{\rho_i} \right) \tag{23}$$

This converges, since $f_n \to \rho_n$ as $n \to \infty$. In practice, for parameter changes that last less than a cell cycle period, this limit converges very rapidly (within a few generations). When the comparison case is simply the eventual behaviour of the cell cycle under the basal parameter set, we have $\rho_i = f_0$, and $f_i = f_0 + \Delta f_i$, giving Equation 14 (Main Text), i.e.:

$$\Delta\phi = \frac{1}{\mu}\ln\left(\prod_{i=1}^{\infty}\frac{f_0 + \Delta f_i}{f_0}\right) \tag{24}$$

This demonstrates that, within the framework of this phenomenological model, there is a strict relationship between changes in the timing of cell cycle events and the fraction of mass donated to the daughter cell upon division.

6.3 Inferring the correspondence between cell cycle characteristics of populations and single cells

In [9], cells were grown under 6 different nutrient limitations, each at 6 different growth rates, and cell cycle characteristics were measured, and presented in the form of population averages. These include the fraction of cells in G1 (denoted F_{G1}), and the average cell size in the population (denoted $\bar{V}$). As discussed in the Main Text, the models consistently predicted that, at constant growth rate, changes in V_{dau} and T_{G1} should be negatively correlated with one another. This prediction was validated under a range of nutrient and genetic perturbations using data from [2], as presented in Figure 2. However, in order to use averaged population data from [9] to provide further validation, a correspondence between the population properties (F_{G1} and $\bar{V}$) and single-cell properties (T_{G1} and V_{dau}) must be found. We begin by relating F_{G1} to T_{G1}. F_{G1} is given by:

$$F_{G1} = \frac{T_{G1,dau}}{T_{div,dau}}F_{dau} + \frac{T_{G1,moth}}{T_{div,moth}}F_{moth} \tag{25}$$

Here, $T_{G1,dau}$, $T_{div,dau}$, $T_{G1,moth}$, and $T_{div,moth}$ denote the durations of the G1 phases, and the durations of the entire cell cycle, in daughter and mother cells, respectively. In

addition, F_{dau} and F_{moth} denote the fractions of daughter and mother cells in the population, respectively ($F_{dau} + F_{moth} = 1$). Noting that mother cells spend little time in G1, we can approximate F_{G1} as:

$$F_{G1} = \frac{T_{G1,dau}}{T_{div,dau}} F_{dau} \tag{26}$$

This demonstrates the expected proportional relationship between $T_{G1,dau}$ and F_{G1} (as has been noted previously [10]).

In the case of the $\bar{V}$, we have:

$$\bar{V} = \bar{V}_{dau} F_{dau} + \bar{V}_{moth} F_{moth} \tag{27}$$

The average daughter cell size is given by:

$$\begin{aligned} \bar{V}_{dau} &= \frac{1}{T_{div}} \int_0^{T_{div}} V_{dau} e^{\mu t} dt \\ &= \frac{V_{dau}}{\mu T_{div}} \left(e^{\mu T_{div}} - 1 \right) \end{aligned} \tag{28}$$

For a mother cell, we assume that they are born at size V_{bud} and produce daughter cells at intervals of $T_{S/G2/M}$ (i.e. their G1 phases are of negligible duration). Furthermore, we approximate these values by their values in daughter cells. Thus, the averaged mother cell size is given by:

$$\bar{V}_{moth} = \frac{1}{T_{S/G2/M}} \int_0^{T_{S/G2/M}} V_{bud} e^{\mu t} dt$$
$$= \frac{V_{bud}}{\mu T_{S/G2/M}} \left(e^{\mu T_{S/G2/M}} - 1 \right)$$
$$= \frac{V_{dau}}{\mu T_{S/G2/M}} \tag{29}$$

References

[1] Ilya Soifer and Naama Barkai. Systematic identification of cell size regulators in budding yeast. *Mol Syst Biol*, 10:761, 2014.

[2] Shivatheja Soma, Kailu Yang, Maria I Morales, and Michael Polymenis. Multiple metabolic requirements for size homeostasis and initiation of division in saccharomyces cerevisiae. *Microbial Cell*, 1(8):256–266, 2014.

[3] Debashis Barik, William T Baumann, Mark R Paul, Bela Novak, and John J Tyson. A model of yeast cell-cycle regulation based on multisite phosphorylation. *Mol Syst Biol*, 6:405, Aug 2010.

[4] Benjamin Pfeuty and Kunihiko Kaneko. Minimal requirements for robust cell size control in eukaryotic cells. *Phys Biol*, 4(3):194–204, Sep 2007.

[5] K. C. Chen, A. Csikasz-Nagy, B. Gyorffy, J. Val, B. Novak, and J. J. Tyson. Kinetic analysis of a molecular model of the budding yeast cell cycle. *Mol Biol Cell*, 11(1):369–391, Jan 2000.

[6] Dorjsuren Battogtokh and John J. Tyson. Bifurcation analysis of a model of the budding yeast cell cycle. *Chaos*, 14(3):653–661, Sep 2004.

[7] D. A. Rand, B. V. Shulgin, D. Salazar, and A. J. Millar. Design principles underlying circadian clocks. *J R Soc Interface*, 1(1):119–130, Nov 2004.

[8] D. A. Rand, B. V. Shulgin, J. D. Salazar, and A. J. Millar. Uncovering the design principles of circadian clocks: mathematical analysis of flexibility and evolutionary goals. *J Theor Biol*, 238(3):616–635, Feb 2006.

[9] Matthew J. Brauer, Curtis Huttenhower, Edoardo M. Airoldi, Rachel Rosenstein, John C. Matese, David Gresham, Viktor M. Boer, Olga G. Troyanskaya, and David Botstein. Coordination of growth rate, cell cycle, stress response, and metabolic activity in yeast. *Mol Biol Cell*, 19(1):352–367, Jan 2008.

[10] Nikolai Slavov and David Botstein. Coupling among growth rate response, metabolic cycle, and cell division cycle in yeast. *Mol Biol Cell*, 22(12):1997–2009, Jun 2011.
